# Computational identification, characterization and validation of potential antigenic peptide vaccines from hrHPVs E6 proteins using immunoinformatics and computational systems biology approaches

**DOI:** 10.1371/journal.pone.0196484

**Published:** 2018-05-01

**Authors:** Abbas Khan, Muhammad Junaid, Aman Chandra Kaushik, Arif Ali, Syed Shujait Ali, Aamir Mehmood, Dong-Qing Wei

**Affiliations:** 1 State Key Laboratory of Microbial Metabolism, and College of Life Sciences and Biotechnology, Shanghai Jiao Tong University, Shanghai, China; 2 Center for Biotechnology and Microbiology, University of Swat, Khyber Pakhtunkhwa, Pakistan; National Chiao Tung University College of Biological Science and Technology, TAIWAN

## Abstract

High-risk human papillomaviruses (hrHPVs) are the most prevalent viruses in human diseases including cervical cancers. Expression of E6 protein has already been reported in cervical cancer cases, excluding normal tissues. Continuous expression of E6 protein is making it ideal to develop therapeutic vaccines against hrHPVs infection and cervical cancer. Therefore, we carried out a meta-analysis of multiple hrHPVs to predict the most potential prophylactic peptide vaccines. In this study, immunoinformatics approach was employed to predict antigenic epitopes of hrHPVs E6 proteins restricted to 12 Human HLAs to aid the development of peptide vaccines against hrHPVs. Conformational B-cell and CTL epitopes were predicted for hrHPVs E6 proteins using ElliPro and NetCTL. The potential of the predicted peptides were tested and validated by using systems biology approach considering experimental concentration. We also investigated the binding interactions of the antigenic CTL epitopes by using docking. The stability of the resulting peptide-MHC I complexes was further studied by molecular dynamics simulations. The simulation results highlighted the regions from 46–62 and 65–76 that could be the first choice for the development of prophylactic peptide vaccines against hrHPVs. To overcome the worldwide distribution, the predicted epitopes restricted to different HLAs could cover most of the vaccination and would help to explore the possibility of these epitopes for adaptive immunotherapy against HPVs infections.

## Introduction

Human papillomaviruses (HPVs), cervical cancer causing agents, are known to be involved in both morbidity and mortality. Annual epidemics of HPV is approximately 0.5 million while the death rate is about 0.25 million worldwide. Many other disorders such as genital, respiratory, warts and hyper proliferative abrasions are associated with these small DNA viruses [1,2]. More than 200 different genotypes of HPVs are characterized. The phylogenetic reconstruction of these genotypes, classified them as Alpha, Beta, Gamma, Mu and Nu. Alpha genus of papillomaviruses are known to be involved in human diseases [3]. Among the characterized species of genus Alpha papillomavirus, most of them are associated with the infection of genital tracts [4,5]. Sexual intercourse is one of the common ways in the transmission of these viruses. However, fomite transmission as a non-major route of transmission has also been reported [6].

High-risk HPVs (hrHPVs) and low-risk HPVs are the two broad categories of HPV Viruses. Out of the total, 99% of cervical cancers are associated with High-risk HPVs (hrHPVs) species (HPV 16, 18, 26, 31, 33, 34, 35, 39, 45, 51, 52, 56, 58, 59, 66, 68 and 70) [7–11]. Among the hrHPVs, HPV16 and 18 are responsible for approximately 75% of the total cases. However, low-risk HPV species (i.e., HPV 6, 7, 11, 32, 42, 43, 44, 54, 61, and 71) are not widely associated with cervical cancer but lead to infection like non-proliferative warts [5,12]. Despite the diversity in pathogenicity, all HPVs shares common genome organization. Core and accessory proteins are the two types of genes products in papillomaviruses. Core proteins, E1 and E2, are reported to be directly involved in the viral replication while L1 and L2 are involved in structural assembly. E4, E5, E6 and E7 are considered as accessory proteins, which show variability in both functional aspects and in expression control. The accessory proteins are reported to be involved in virus replication inside infected cell. E6 and E7, two important oncoproteins, are found to be expressed in all positive cases of cervical cancer and are responsible for viral entry, cellular alteration and tumor induction [13–16]. Experimental results proved that the expression of E6 and E7 proteins is the primary cause of the immortalization of primary human keratinocytes in a genome wide study [17–19]. Beside the carcinogenesis, the deactivation of the tumor suppressor proteins, such as p53 and the retinoblastoma (pRb), is due to the continuous expression of E6 proteins in the cellular environment. Interaction of E6 proteins with E6AP alter the substrate specificity substrate specificity and polyubiquitylates p53, leading to the in degradation of p53 aided by 26S proteasome [20,21]. Therefore, the important role of E6 in causing and developing cervical cancer is important and clear. On the other hand, E7 protein perform the function of degradation of pRb and p130 which is a proteasome-dependent process [12,22].

Prediction and development of novel vaccine candidates against the complex diseases has sophisticatedly provoked the desired response and has greatly aided the work of molecular and chemical biologists to expose safe and effective vaccines [23]. Immunological mechanisms of surface presentation of antigen along with MHC protein directs the activation of cytotoxic T-lymphocyte (CTL), being an effector, to kill the infected cell. Upon the interaction of CTLs on the infected cell, self-destruction or apoptosis is observed typically. Usually the peptide fragment of the pathogen confers this signaling process and thus provoke the immunity. The underlying mechanism is the attachment of peptide fragment, usually a virulent factor, binds to the MHC molecule and is presented on the surface of infected cells. This process rely on the proteasomal cleavage and transportation to the endoplasmic reticulum (ER) along with MHC molecule. The antigen processing channels (TAP) are required to present the peptide-MHC complex on the surface of the cell for immune response. Therefore, considering the c-terminal cleavage activity and TAP efficiency greatly help in the selection of effective vaccine candidates [24–27].

The purpose of our present study is to promote the designing of a vaccine against hrHPVs species using in silico methods, taking the most important protein E6 into consideration. The important role of this protein in the cervical cancer carcinogenesis is clear. Therefore, we designed an epitope-based peptide vaccine against hrHPVs that are important but neglected by most of the researchers. To date only HPV 16 & 18 are studied for immunological studies. To fill this gap and study other hrHPVs this meta-analysis was carried out to predict antigenic potential peptide vaccines using immunoinformatics approach.

## Materials and methods

### E6 protein sequences

**Fig 1:**
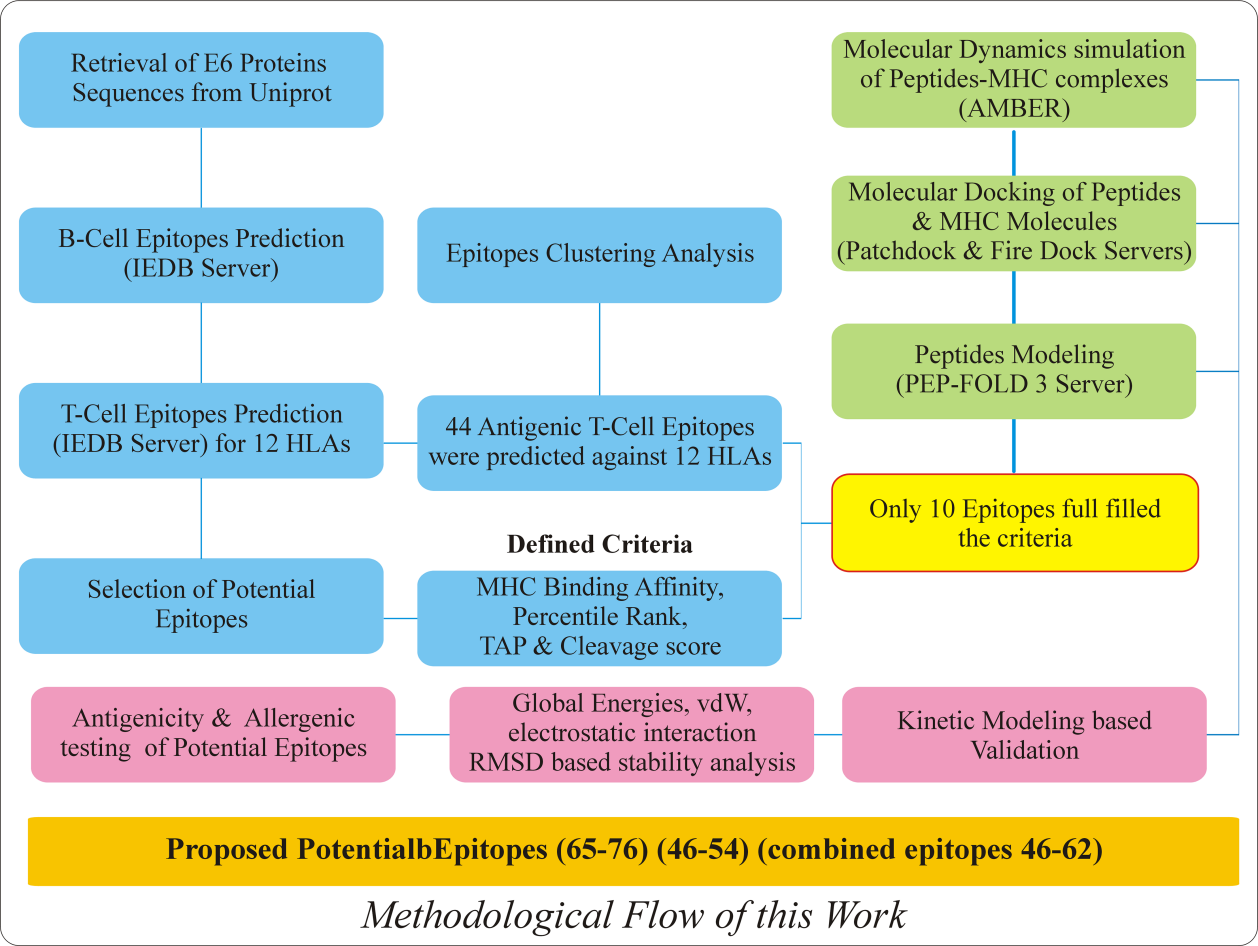
Schematic of the workflow for the prediction and validation of peptide vaccines from hrHPVs E6 proteins. The above figure is showing the whole methodology including the resources and results obtained from these analyses.

The primary amino acid sequences of hrHPVs (HPV31, HPV33, HPV35, HPV39, HPV45, HPV51, HPV52, HPV56, HPV58, HPV68) E6 Proteins were retrieved from Universal Protein Resources (Uniprot) (http://www.uniprot.org/). The detail information including accession number, protein sequence length and species used are given in the S1 Table. The schematic flow of this work is given in the Fig 1.

### Prediction of linear B-cell epitopes

Interaction of B-lymphocytes with antigen B-cell epitope directs the differentiation of B-lymphocytes into memory cells and antibody secreting-plasma [28]. The characteristics properties such as accessibility for flexible region and hydrophilic nature are important for B-cell epitopes [29]. Different *in silico* peptide development approaches such Parker hydrophilicity prediction [30], Emini prediction of surface accessibility [31], Kolaskar and Tongaonkar’s antigenicity [32], Karplus and Schulz Flexibility Prediction were used using an online analysis resource at IEDB (http://www.iedb.org/). ElliPro [33] (http://tools.immuneepitope.org/toolsElliPro/) is an integrated tool in an online IEDB server which can predict B-cell epitopes using both structural information or protein sequences. ElliPro employ three different algorithms including Protrusion Index (PI) of residues, protein shape approximation and the final neighboring residues clustering which rely on PI.

### Prediction of potential cytotoxic T-lymphocyte (CTL) epitopes

NetCTL.1.2 [34] (http://www.cbs.dtu.dk/services/NetCTL/) is an online most widely using server for the prediction of CTL epitopes. The Proteomic data from all the hrHPVs were screened to predict potential T-CD8+ (MHC class I binding epitopes) epitopes by using algorithms NetCTL and NetMHC [35,36]. NetCTL accept FASTA sequence as an input that perform different analysis such as prediction of MHC class I binding affinity, TAP transport efficiency and C-terminal Cleavage activity. Concerning the MHC alleles, the predictions were restricted to 12 human alleles HLA-A*0101, HLA-A*0201, HLA-A*2402, HLA-A*2601, HLA-B*0801, HLA-B*2705, HLA-B*3901, HLA-B*4001, HLA-B*501, HLA-B*1501, HLA-C*0801 and HLA-C*0202. The weight matrix and artificial neural network was used for the prediction of MHC-I binding and proteasome C-terminal cleavage.

### Allergenic and antigenic profiling of B & T-cell predicted epitopes

In order to validate the non-allergenic potential of the predicted B-cell and T-cell epitopes an online web tool AlgPred [37] (http://crdd.osdd.net/raghava/algpred/) was utilized by using multiple algorithms (SVMc, IgEepitope, ARPs BLAST and MAST) to predict the allergenic peptides with an accuracy of 85% by combining these methods. Therefore, we used the primary amino acid sequences to test the allergenic potential of all the selected E6 proteins. On the other hand, to map the antigenic index of our predicted epitopes ANTIGENpro [38] (http://scratch.proteomics.ics.uci.edu/) was used. This server access five different machine-learning algorithms and multiple representation of primary sequences to pile up the antigenicity results by protein microarray data analysis.

### Peptides libraries construction and molecular docking

The 3D coordinates of all the selected peptides were predicted by using an online effective web server PEP-FOLD3. For sampling the conformations of predicted peptides simulation runs was set 200 [39]. sOPEP energy function integrated in PEP-FOLD3 was applied to cluster the diverse conformational models [40]. Selection of specific epitope from all the species was based on low percentile rank and high C-terminal cleavage activity with good TAP score. Sharing of amino acids between the B-cell epitopes and T-cell epitopes were also selected as a docking criteria. Afterward, the best peptide coordinates were docked to the class I MHC molecules HLA-A*0101 **(PDB ID 4NQV)**, HLA-B*1501 **(PDB ID 1XR9)**, HLA-B*5801 **(PDB ID 5IM7)** and HLA-C*0801 **(PDB ID 4NT6)** using the PatchDock rigid-body docking server based on the defined threshold [41]. PatchDock uses a geometry based docking algorithm to find docking transformations with good molecular shape complementarity [42]. Scoring and refining of the docked complexes produced by fast rigid-body docking was performed by employing FireDock server [43,44]. Complexes with high global docking energy, Attractive Vander Waal Energy and Hydrogen Bonding energy were subjected to molecular dynamics simulation [45].

### Kinetics simulation for the validation of predicted epitopes

A computational systems biology workbench [46] was used to design and execute an *in silico* biochemical pathway to confirm the antigenic potential of the peptides. Kinetics simulation or pharmacokinetics simulation [47] is useful tool to describe the sufficient dose of a testing drug. The pharmacokinetics of most drugs is first order at therapeutic doses. This non-linear kinetics scheme follow simple Mass kinetics equation or Typically, Michaelis–Menten equation [48] as shown below;

$$V=\frac{\left(Vmax).(S\right)}{Km+S}$$ (i)

This equation can be transformed to

$$C=\frac{\left(Cmax).(D\right)}{Km+D}$$

Or

$$V=\frac{d[P]}{dt}=\frac{\left(Amax).(D\right)}{Km+D}$$ (ii)

Here C represents the steady state concentration, C_max_ the theoretical maximum for C, A the amount absorbed, A_max_ the theoretical maximum for A, and D the dose.

The literature survey was done to collect the necessary information for the hrHPVs. A pharmacokinetics pathway was established to validate the peptides for their potential action. Nodes in the pathway represent the entities, and edges represent the connectivity of one node to another node, which is closely related to each other. In order to carry out pharmacokinetic, concentration doses (0.2 μm) were assigned from available research [49].

### Epitope cluster analysis

Clustering of epitopes into groups based on identity among the selected proteins sequences was carried out with the aid of an online Epitope Cluster Analysis tool (http://tools.immuneepitope.org/main/index.html). In the current study, a cluster is a group of sequences sharing a minimum of 80% of the sequence identity is known to be a cluster.

### Molecular dynamics simulations

MD simulations of all the selected complexes were carried out by using AMBER 14 molecular dynamics package [50]. To neutralize the systems counter Na+ ions and hydrogens were added. The tleap package of Amber was utilized to perform this process. A TIP3P water box of 8.0 A° was used. A two stages energy minimization, each of 6000 steps, of the complexes using the SANDER module of AMBER 14, was performed to remove the constraints all atoms in the systems except those from the water molecules. PMEMD.cuda [51] unit of AMBER 14 was used to accomplish MD simulations of the minimized complexes. For long-term interactions, the SHAKE algorithm and Particle-Mesh Ewald (PME) method was used and a non-bond contacts cutoff radius of 10A° was kept. Using the Langevin temperature 310K and constant pressure (1atm) with isotropic molecule-based scaling was considered for equilibration of 10,000 ps time, followed by a total of 20ns simulation was carried. Sampling of MD trajectories was carried out after every 2.0 ps time scale. Analysis such as RMSD and Hydrogen bonding analysis was carried out by using an integrated programs CPPTRAJ and PYTRAJ [52] in AMBER 14. The following equation was solved to calculate the stability of the complexes after 20ns.

$$RMSD=\sqrt{\frac{\sum_{i=0}^{N}\left[m_{i}*(X_{i}*Y_{i}{)}^{2}\right]}{M}}$$

Where N is the number of atoms, m_*i*_ is the mass of atom *i*, x_*i*_ is the coordinate vector for target atom *i*, Y_*i*_ is the coordinate vector for reference atom *i*, and M is the total mass. If the RMSD is not mass-weighted, all m_i_ = 1 and M = N.

### Hydrogen bonding analysis

Hydrogen bonds are an important non-covalent structural force in molecular systems. They are formed when a single hydrogen atom is effectively shared between the heavy atom it is covalently bonded to (the hydrogen bond donor) and another heavy atom (the hydrogen bond acceptor). Here, we analyzed the hydrogen bonds between all the selected complexes. Hydrogen Bonds were analyzed at three different stages. The bonds were checked before the simulation and after the simulation. After the minimization and production in the PDB coordinated of the complexes were saved from the .rst files and were analyzed by using an online server PDBePISA [53] UCSF Chimera [54] and PyMOL [55] visualization software.

## Results

### Antigenic B-cell epitopes prediction

The antigenic epitopes were determined by using Tongaonkar’s method [32] using the physiochemical properties of amino acids. Experimental precision for this method is observed to be 75% [32]. Four antigenic peptides in each E6 protein of HPV31, HPV35, HPV45, and HPV68 were predicted that range from 7–15 amino acids. Moreover, HPV39, HPV51, HPV52 and HPV58 possess five antigenic epitopes, ranging from 6–14 amino acids respectively. HPV56 contain three antigenic epitopes while HPV33 possess six antigenic epitopes. The range of HPV56 epitopes is 9–14 amino acids while HPV33 peptides range from 6–14 amino acids. The B-cell epitopes predicted are shown in the Table 1.

**Table 1:** Predicted antigenic B-cell epitopes of hrHPVs E6 proteins. A total of 44 antigenic sites were identified from all the E6 proteins. Residues shared by both B-cell and T-cell epitopes are given in bold.

Specie	No	Start	Stop	Peptide Sequence	Length
**HPV31**	1	1	13	MFKNPAERPRKLH	13
	2	36	42	QLTETVL	7
	3	109	120	PLCPEEKQRHLD	12
	4	1	13	MFKNPAERPRKLH	13
**HPV33**	1	1	11	MFQDTEEKPRT	11
	2	35	42	KPLQRSEV	8
	3	55	60	REGNPF	6
	4	82	95	SVYGNTLEQTVKKP	14
	5	110	119	LCPQEKKRHV	10
	6	130	137	GRWAGAAC	8
**HPV35**	1	1	11	MFQDPAERPYK	11
	2	56	61	EGQPYG	6
	3	83	91	VYGETLEKQ	9
	4	110	120	LCPVEKQRHLE	11
**HPV39**	1	4	15	FHNPAERPYKLP	12
	2	38	44	PLQQTEV	7
	3	57	64	RDGEPLAA	8
	4	83	88	DSVYAT	6
	5	132	137	GSYTGQ	6
**HPV45**	1	2	16	ARFDDPKQRPYKLPD	15
	2	39	45	LERTEVY	7
	3	83	93	NSVYGETLEKI	11
	4	110	122	KPLNPAEKRRHLK	13
**HPV51**	1	1	11	MFEDKRERPRT	11
	2	55	61	RDNNPYA	7
	3	81	86	RSVYGT	6
	4	107	120	QRPLGPEEKQKLVD	14
	5	130	135	GRWTGQ	6
**HPV52**	1	1	11	MFEDPATRPRT	11
	2	36	42	ELQRREV	7
	3	55	60	RDNNPY	6
	6	86	96	KTLEERVKKPL	11
	7	110	121	LCPEEKERHVNA	12
**HPV56**	1	1	14	MEPQFNNPQERPRS	14
	2	86	94	VYGATLESI	9
	3	110	121	QSPLTPEEKQLH	12
**HPV58**	1	2	11	FQDAEEKPRT	10
	2	36	41	TLQRSE	6
	3	55	60	RDGNPF	6
	4	83	90	LYGDTLEQ	8
**HPV68**	1	5	14	HNPEERPYKL	10
	2	59	65	GVPFAAC	7
	3	83	88	ESVYAT	6
	4	91	96	ETITNT	6

To predict the maximum residual score for each amino acid in the E6 of hrHPVs species Kolaskar and Tongaonkar’s was used. Proteins with residual score >1 were quantified. Among the total selected proteins large number of residues with score greater than 1 were found, which is showing the antigenic potential of E6 protein. The graphical illustration, given in S1 Fig, of predicted antigenic propensity, maximum and minimum residual score and number of residues with residual score >1 are given in the S2 Table.

### Surface accessibility for E6 proteins

The surface probability of each residue was predicted using a threshold 1.0. Amino acids with the score greater than 1 has the highest probability to be found on the surface [31]. The minimum surface probability score 0.05 for HPV31 from amino acid position (ICDLLI_96-101_), 0.032 for HPV33 from amino acid position (IRCIIC_101-106_), 0.034 for HPV35 from amino acid position (ICLNCV_26-31_), 0.035 for HPV39 from amino acid position (IRCMCC_103-108_), 0.032 for HPV45 from amino acid position (IACVYC_30-35_), 0.025 for HPV51 from amino acid position (VVCVYC_28-33_), 0.021 for HPV52 from amino acid position (VCIMCL_62-67_), 0.041 for HPV56 from amino acid position (VCRVCL_65-70_), 0.003 for HPV58 from amino acid position (IRCIIC_101-106_) and 0.065 for HPV68 from amino acid position (IDCVYC_30-35_) was calculated as the surface accessibility score. On the other hand, the maximum surface probability score 5.234 (PEEKQR_112-117_), 6.016 (PQEKKR_112-117_), 5.15(KPTRRE_141-146_), 7.349 (KREDRR_145-150_), 5.289(RRRRET_151-156_), 5.536 (KRERPR_5-10_), 5.353 (PEEKER_112-117_), 4.74 (RKYRYY_77-82_), 6.653 (RPRRRQ_141-146_) and 7.205 (KREDRR_145-150_) was predicted for all HPVs from HPV31 to HPV68. S2 Fig is showing the graphical illustration of predicted Surface accessibility of E6 Proteins of hrHPVs.

### Surface flexibility for E6 proteins

Temperature or B factor is used to demonstrate the back and forth motion of atoms within a protein coordinates. To calculate the motion of atoms Karplus and Schulz’s flexibility method was implemented. Atoms with profoundly systematized structure appeared to have low B-factor while the distorted appeared higher [56]. HPV31, HPV35, HPV52 and HPV56 showed a minimum flexibility score of 0.901 for heptapeptides LIRCITC_100-106_, LLIRCIT_99-105_, QVVCVYC_27-33_ and RLSCVYC_30-36_ respectively. The rest HPV33, HPV39, HPV45, HPV51, HPV58 and HPV68 showed a minimum uniform score of 0.885 to 0.889 for more ordered structure of heptapeptides LIRCIIC_100-106_, LIRCMSC_102-108_, CIAYAAC_59-65_, VCIMCLR_62-68_, LIRCIIC_100-106_ and LIRCMSC_102-108_ respectively. On the other hand, maximum surface flexibility score for E6 proteins from HPV31-68 showing a more ordered structure with a sequence of heptapeptides PEEKQRH_112-118_, CPQEKKR_111-117_, YREGQPY_54-60_, YRDGEPL_56-62_, DDPKQRP_5-11_, PEEKQKL_112-118_, PEEKERH_112-118_, RQTSREP_144-150_, CPQEKKR_111-117_ and RIRQETQ_151-157_ were found 1.089, 1.088, 1.091, 1.067, 1.084, 1.095, 1.076, 1.101, 1.088 and 1.077 respectively. S3 Fig is showing the graphical representation of predicted Surface flexibility of E6 Proteins of hrHPVs.

### Parker hydrophilicity prediction for E6 protein

Hydrophilicity of the predicted peptide was calculated based on retention times of a peptide during HPLC using reversed phase column. Here, we used Parker hydrophilicity prediction method to predict the water loving potential of the predicted antigenic peptides. Immunological studies reported the direct association of hydrophilic region with the antigenic sites [30]. S4 Fig is showing the graphical illustration of predicted Parker Hydrophilicity of E6 Proteins of hrHPVs on the basis of the x-axis is showing the position of the amino acids and y-axis is plotting the hydrophilicity. Among the selected species, the lowest hydrophilicity was calculated as -5.086 from all the E6 protein of HPV35 and HPV58 from amino acid position LCHLLIR_96-102_ and ILIRCII_99-105_. These regions were predicted to act as active T-cell epitopes. On the other hand the maximum hydrophilicity score 6.371 was calculated for HPV35 E6 protein for the peptide sequence QDTEEKP_3-9_. Moreover, the maximum and minimum hydrophilicity score for all the other HPV species are shown in the S3 Table.

### Antigenic T-cell epitopes prediction

Cytotoxic T-lymphocyte (CTL) epitopes were explored from the E6 proteins of the selected hrHPVs. NetCTL 1.2 server [34] was utilized to predict the CTL epitopes. MHC binding affinity, proteasomal C-terminal cleavage, TAP transport affinity and potential MHC ligands were recognized by following > 0.75000 threshold as criteria. A total of 38 peptide sequences from E6 Proteins of all the selected hrHPVs were predicted as CTL epitopes whose prediction score were > 0.75000 (Table 2).

**Table 2:** Predicted CTL epitopes from (HPV31 to HPV68) E6 protein. **Prediction score threshold was set at > 0.75000.** Bold indicates amino acids that were also predicted as antigenic sites.

Residue No	Peptide Sequence	MHC Binding Affinity	Rescale Binding Affinity	C-terminal Cleavage Affinity	Transport Affinity	Prediction Score	MHC-I Binding
**15**	LSSALEIPY	0.4620	1.9614	0.6850	2.7980	2.2040	Yes
**37**	**LTETE**VLDF	0.5417	2.3000	0.8056	2.3240	2.5371	Yes
**39**	**ETE**VLDFAF	0.2203	0.9355	0.0724	2.1750	1.0551	Yes
**47**	FTDLTIVYR	0.1915	0.8130	0.8104	1.3460	1.0019	Yes
**73**	**VSEFRWYRY**	0.5615	2.3842	2.3842	2.9840	2.6666	Yes
**39**	**RSEV**YDFAF	0.2390	1.0147	0.1258	2.6260	1.1649	Yes
**73**	ISEYRHYNY	0.5954	2.5278	0.8555	2.9450	2.8033	Yes
**2**	**FQDPAERPY**	0.2014	0.8549	0.8643	2.7650	1.1228	Yes
**73**	ISEYRWYRY	0.5572	2.3657	0.8670	2.9450	2.6430	Yes
**41**	**QTEV**YEFAF	0.3068	1.3027	0.0842	2.3330	1.4320	Yes
**45**	YEFAFSDLY	0.1254	0.5323	0.9668	2.9280	0.8237	Yes
**49**	FSDLYVVYR	0.1983	0.8419	0.8143	1.4080	1.0345	Yes
**72**	YAKIRELRY	0.2013	0.8546	0.9407	2.8500	1.1382	Yes
**81**	YS**DSVYAT**T	0.3351	1.4228	0.0616	0.9130	1.3863	Yes
**95**	NTKLYNLLI	0.1752	0.7439	0.7623	0.5580	0.8862	Yes
**18**	CTELNTSLQ	0.2302	0.9776	0.0277	-0.2640	0.9686	Yes
**37**	AT**LERTEVY**	0.3216	1.3654	0.9669	3.1660	1.6687	Yes
**41**	**RTEVY**QFAF	0.3261	1.3848	0.2831	2.4460	1.5495	Yes
**72**	YSRIRELRY	0.3333	1.4151	0.8846	2.8890	1.6922	Yes
**95**	NTELYNLLI	0.4589	1.9483	0.8040	0.4160	2.0897	Yes
**39**	RADVYNVAF	0.2034	0.8635	0.3223	2.6480	1.0442	Yes
**89**	EAITKKSLY	0.1378	0.5852	0.9040	2.8900	0.8653	Yes
**47**	FTDLRIVYR	0.1636	0.6948	0.5907	1.2400	0.8454	Yes
**73**	ISEYRHYQY	0.5593	2.3746	0.9645	2.9450	2.6666	Yes
**76**	YRHYQYSLY	0.1668	0.7084	0.9696	2.9740	1.0025	Yes
**73**	YSKVRKYRY	0.3031	1.2871	0.8705	2.8380	1.5596	Yes
**99**	LCDLLIRCY	0.1708	0.7250	0.8642	2.7240	0.9909	Yes
**35**	K**TLQRSE**VY	0.2565	1.0890	0.8691	3.1290	1.3758	Yes
**39**	**RSEV**YDFVF	0.2152	0.9138	0.2751	2.6260	1.0864	Yes
**68**	RLLSKISEY	0.1342	0.5698	0.9665	3.2540	0.8774	Yes
**73**	ISEYRHYNY	0.5954	2.5278	0.9588	2.9450	2.8188	Yes
**76**	YRHYNYSLY	0.1608	0.6829	0.9673	2.9740	0.9767	Yes
**20**	TLDTTLHDV	0.1793	0.7613	0.7970	0.2690	0.8943	Yes
**41**	RTEVYEFAF	0.2257	0.9584	0.1397	2.5640	1.1075	Yes
**49**	FSDLCVVYR	0.1559	0.6619	0.2967	1.4080	0.7768	Yes
**72**	YAKIRELRY	0.2013	0.8546	0.9002	2.8500	1.1322	Yes
**91**	**ETITNTKLY**	0.3233	1.3728	0.8601	2.8160	1.6426	Yes
**95**	NTKLYNLLI	0.1752	0.7439	0.8751	0.5580	0.9031	Yes

### Molecular docking of E6 proteins with HLA-A*0101

**Fig 2:**
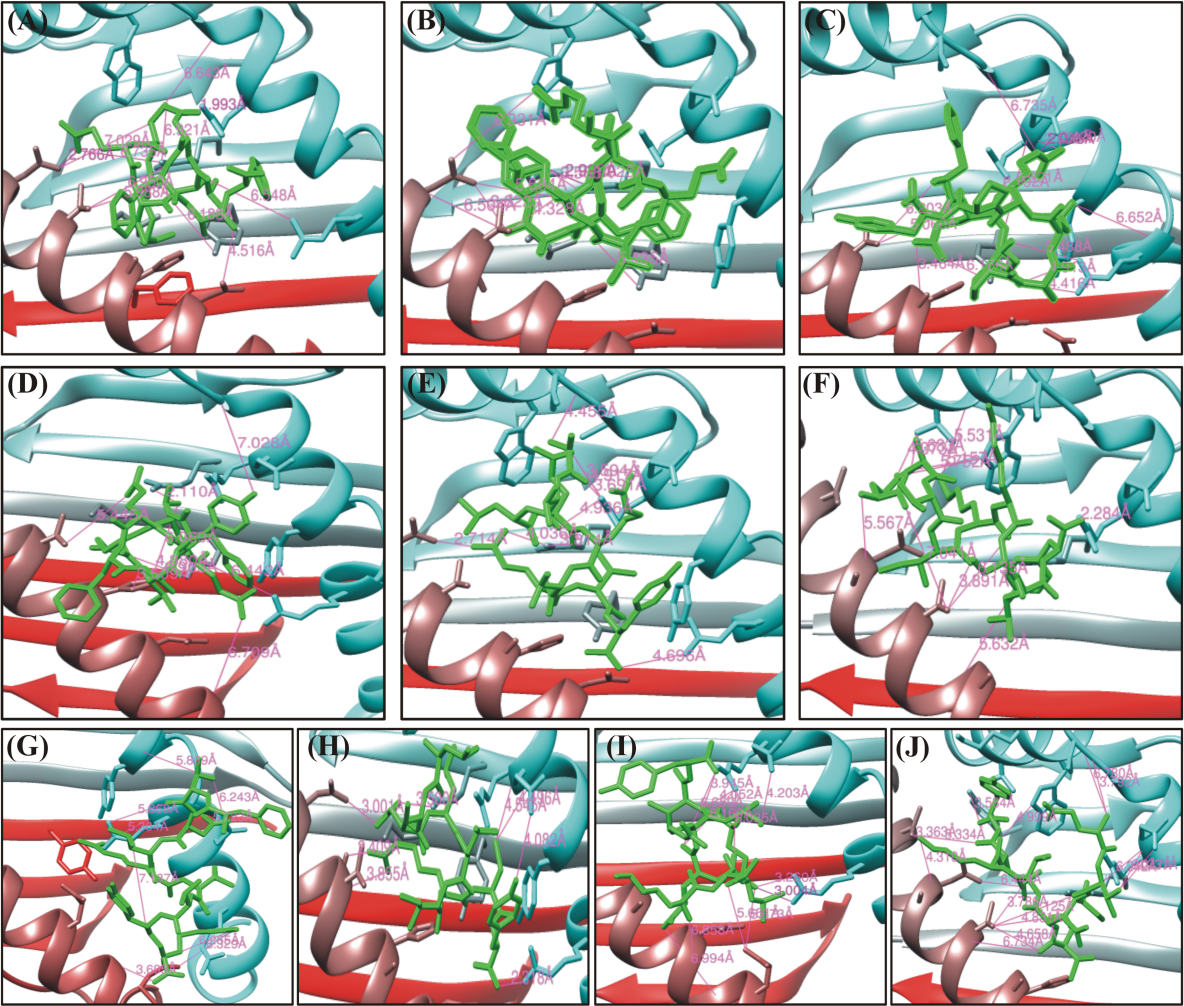
Molecular interaction analysis of predicted HPVs E6 peptides docked to MHC-I HLA-A*0101. (A) ETEVLDFAF, (B) RSEVYDFAF, (C) FQDPAERPY, (D) QTEVYEFAF, (E) ATLERTEVY, (F) EATIKKSLY, (G) FTDLRIVYR, (H) LCDLLIRCY, (I) KTLQRSEVY, (J) ETITNTKLY.

Among the total 38 epitopes, only 9 epitopes, were docked to MHC class I HLA-A*0101, 2 epitopes against the HLA-B*1501, 1 epitope against HLA-B*5801 and 3 epitopes against HLA-C*0801 from all the selected HPV species as shown in the Table 3. Initially, epitopes predicted against the 12 human alleles with good percentile rank, TAP transport efficiency and C-terminal Cleavage activity were selected to be analyzed for the binding efficiency [57] approach. A 2% percentile was used in the present study. Because it has been reported that using a defined threshold of percentile rank and MHC binding affinity, most of the predicted epitopes provoked the immune response in experimental condition. The global and attractive van der Waals energy (vdW) were computed ranging from -22.25 to -54.31 kcal/mol and -17.52 to -31.80 to determine the binding efficiency of each epitope respectively. The docking scores along with the bonding pattern is presented in the Table 4. Among the essential features Asn77, Tyr99, Arg114 and Arg156 residues from the MHC-I groove were most abundantly involved in bonding with different predicted peptides. Among the total 10 epitopes docked against HLA-A*0101 (ETEVLDFAF, RSEVYDFAF and KTLQRSEVY) showed the highest binding affinities and highest number of hydrogen bonding within 3Å. However, the other epitopes also showed good affinities. This analysis established a good interaction of the modeled antigenic peptides with the MHC-I molecules. Nevertheless, residues shared by MHC-I in bonding with different peptides are also reported by *Mirza*, *Rafique et al*. [58] in the same computational study while predicting antigenic epitopes. Furthermore, residues from different epitopes such as (Arg5, Glu3 and Thr4) were predicted to act as antigenic. The graphical representation of these docked complexes are given in the Fig 2. The hydrogen bonds with length less than 3Å were most frequently found in all complexes. Overall stability of the docked complex seems to be well preserved by the formation of hydrogen bonds.

**Table 3:** Binding affinity against each allele is determined in terms of percentile rank by IEDB for MHC class I. Based on the low percentile rank (2%) these epitopes were docked against the specific allele.

Allele	Length	Peptide	Method used	Percentile rank
HLA-A*0101	9	ETEVLDFAF	Consensus (ann/smm)	0.8
HLA-A*0101	9	RSEVYDFAF	Consensus (ann/smm)	0.95
HLA-A*0101	9	FQDPAERPY	Consensus (ann/smm)	1.0
HLA-A*0101	9	QTEVYEFAF	Consensus (ann/smm)	0.75
HLA-A*0101	9	ATLERTEVY	Consensus (ann/smm)	0.8
HLA-A*2601	9	ATLERTEVY	Consensus (ann/smm)	1.45
HLA-B*1501	9	ATLERTEVY	Consensus (ann/smm)	1.9
HLA-A*2601	9	EATIKKSLY	Consensus (ann/smm)	0.75
HLA-A*0101	9	FTDLRIVYR	Consensus (ann/smm)	0.65
HLA-A*0101	9	LCDLLIRCY	Consensus (ann/smm)	0.65
HLA-B*5801	9	KTLQRSEVY	Consensus (ann /smm)	0.9
HLA-B*1501	9	KTLQRSEVY	Consensus (ann /smm)	1.3
HLA-A*0101	9	KTLQRSEVY	Consensus (ann/smm)	1.3
HLA-A*2601	9	ETITNTKLY	Consensus (ann/smm)	0.1
HLA-A*0101	9	ETITNTKLY	Consensus (ann/smm)	0.55
HLA-C*0801	9	FTDLRIVYR	Consensus (ann/smm)	0.4
HLA-C*0801	9	FQDPAERPY	Consensus (ann/smm)	0.6
HLA-C*0801	9	ATLERTEVY	NetMHCpan	1.8

**Table 4:** HPV E6 peptides–HLA-A*0101 interaction. FireDock energy for the best ranked complex initial distance between the H-bond donor and the acceptor; measured with the Find H.Bond tool in Chimera (H-Bond constraints were relaxed by 1 Å and 20.0 degrees) distance between the H-bond donor and the acceptor after molecular dynamics simulation (MD); measured in PyMOL, nd = no detected H-bond.

Peptide	Global Energy (kcal/mol)	vdW energy (kcal/mol)	H-Bond energy (kcal/mol)	Atomic interactions	
				Peptide-MHC atom pair	d_init_ (Å)
**ETE**VLDFAF	**-41.89**	**-26.62**	**-2.13**	Arg156 NH1-Asp6 OD1	1.99
**HPV31**				Arg156 NH2-Asp6 OD2	2.90
				Asn77 OD1—Arg5 NH1	2.74
				Arg114 NH2-Thr2 OG1	2.74
				Asn77 ND2-Glu3 OE1	2.77
				Arg114 NH2-Glu3 OE1	6.7
				Asn77 ND2-Glu3 OE1	2.76
**RSEV**YDFAF	**-54.31**	**-31.80**	**-3.05**	Thr73 OG1-Tyr5 O	4.32
**HPV33**				Asn77 ND2-Ala8 O	3.62
				Arg114 NH1-Phe9 O1	3.56
				Arg114 NH2-Asp6 O	1.92
				Arg114 NH2-Phe9 O1	2.99
				Arg163 NH1-Glu3 O	4.41
				Asn77 ND2-Ala8 O	3.62
				Arg156 NH2-Asp6 OD1	3.61
				Arg156 NH2-Asp6 OD2	2.36
**FQDPAERPY**	**-34.85**	**-22.04**	**-2.13**	Gln2 NE2-Ala69 O	3.46
**HPV35**				Arg7 NE-Gln155 OE1	2.04
				Arg7 NH2-Gln155 OE1	2.15
				Arg7 NH2-Gln155 O	2.42
				Tyr9 OH-Asn77 OD1	5.93
				Gln2 O -Arg156 NH1	2.19
				Tyr99 OH-Asp3 OD1	3.16
				Ala69 O-Gln2 NE2	3.46
				Thr73 OG1-Gln2 NE2	2.92
				Arg156 NH1-Gln2 O	2.19
**QTEV**YEFAF	**-37.08**	**-21.66**	**-2.72**	Asn77 ND2-Glu3 OE2	6.20
**HPV39**				Asn77 ND2-Glu6 OE1	6.26
				Tyr99 OH -Glu3 OE1	4.88
				Arg11 NH2-Thr2 OG1	2.11
				Arg163 NH1-Gln1 OE1	2.33
				Asn66 OD1 -Gln1 NE2	3.29
				Gln155 OE1-Tyr5 OH	2.24
				His70 ND1 -Glu3 OE1	2.98
				His70 NE2 -Glu3 OE1	2.74
AT**LERTEVY**	**-35.84**	**-24.74**	**-2.50**	Arg114 NH2-Glu4 O	3.81
**HPV45**				Arg156 NH1-Thr2 O	5.57
				Arg156 NH2-Ala1 O	3.69
				Arg156 NH2-Thr2 O	4.93
				Arg156 NH2-Ala1 O	3.13
				Arg114 NH2-Glu4 O	3.69
				Arg156 NH2-Glu4 OE1	3.81
				His70 NE2-Glu7 O	3.59
				Asn77 ND2-Glu7 OE2	2.76
				Tyr99 OH -Thr6 O	2.71
				Arg114 NE -Glu4 O	5.64
FTDLRIVYR	**-28.53**	**-21.66**	**-3.93**	Val158 O-Thr2 OG1	5.88
**HPV52**				Asn66 OD1-Arg5 NE	4.69
				Arg163 O-Arg5 NH1	5.30
				Asn66 OD1-Arg5 NH2	4.40
				Tyr99 OH -Arg5 NH2	4.07
				Tyr59 N-Arg9 O1	3.69
				Arg163 NH1-Thr2 O	5.86
				Asp166 N-Asp3 OD1	5.72
				Tyr159 OH-Arg5 NH1	2.49
				Arg163 N-Asp3 OD1	3.04
				Glu63 OE2-Arg5 NE	3.75
LCDLLIRCY	**-32.62**	**-21.66**	**-2.32**	Asp74 OD1-Arg7 NH2	2.40
**HPV56**				Gln155 OE1-Tyr9 OH	4.08
				Tyr99 OH -Ile6 O	5.40
				Arg163 NH2-Tyr9 O2	2.31
				Arg156 NH2-LEU1 O	3.12
				Asn77 ND2-Asp3 OD1	3.00
				Arg114 NH2-Asp3 OD2	3.50
				Arg163 NH1-Cys8 O	2.88
				His70 O-Arg7 NH2	3.75
				Asp74 OD1-Arg7 NH2	2.41
				Arg114 NH2-Asp3 OD2	3.50
				Asp74 OD1-Arg7 NH1	3.53
K**TLQRSE**VY	**-40.59**	**-26.79**	**-2.66**	Arg65 O-Gln4 NE2	2.17
**HPV58**				Arg163 NH2-Glu7 OE2	3.00
				Arg163 NH1-Glu7 OE2	3.26
				Arg163 NH2-Glu7 OE2	3.00
				Arg65 NE -Gln4 OE1	5.87
				Thr73 OG1-TYR9 OH	5.41
				Arg114 NE -Glu7 O	4.95
				Arg114 NH2-Glu7 O	4.42
				Arg156 NH1-Leu3 O	6.03
				Arg156 NH1-Gln4 O	6.16
				Arg156 NH1-Arg5 O	4.05
				Arg156 NH2-Arg5 O	3.94
				Gln155 OE1-Ser6 OG	4.20
ETITNTKLY	**-28.18**	**-24.68**	**-1.90**	Arg156 NH2-Glu1 O	2.11
**HPV68**				Thr73 OG1-Thr6 O	3.79
				ASN77 OD1-Tyr9 N	3.74
				Gln155 O -Thr2 OG1	6.04
				Gln155 OE1-Thr2 OG1	4.23
				Tyr99 OH -Thr4 OG1	5.21
				Tyr123 OH -Tyr9 O1	3.58
				Trp147 NE1-Lys7 O	4.99
				Arg163 NH1-Thr4 OG1	5.77

### Molecular docking of E6 proteins with HLA-B*1501 and HLA-B*5801

**Fig 3:**
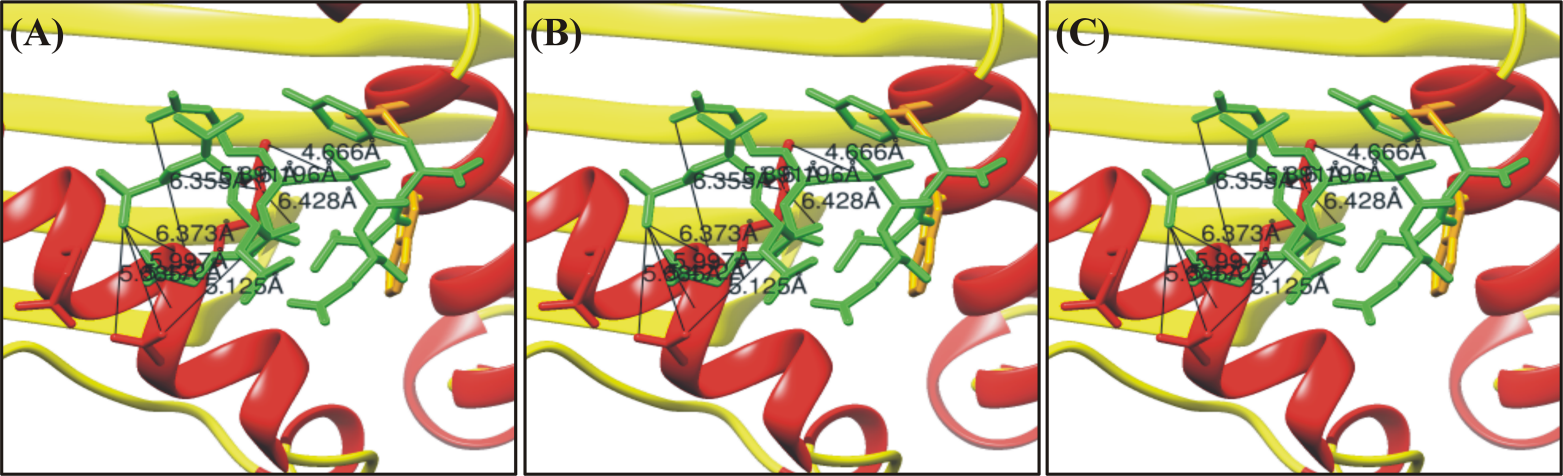
Molecular interaction analysis of predicted HPVs E6 peptides docked to MHC-I HLA-B*1501 and HLA-B*5801. Epitope A and B (ATLERTEVY, KTLQRSEVY) were docked against HLA-B*1501 while epitope C (KTLQRSEVY) shown in the figure above was docked against HLA-B*5801.

Epitope ATLERTEVY was found to be good against HLA-B*1501 with the percentile rank 1.9. While epitope KTLQRSEVY with percentile rank 1.3 and 0.9 restricted to both HLA-B*1501 and HLA-B*5801 showed good affinity. The global energies of each of these epitopes restricted specific allele were -30.47, -24.89 and -21.19 kcal/mol while the attractive van der Waals energy (vdW) were ranging from -18.84 to -20.29 kcal/mol respectively. Asp61, Tyr59, Asn66, Arg97 and Ser73 from these three MHC-I molecules were frequently in vigorous interaction with the predicted peptides. Complexes after the docking were subjected to analyze the hydrogen bonding within 3Å. Variable hydrogen bonds (2 to 4) within 3Å were found. Molecular interactions of the specific peptides and their respective docked poses are tabulated in Table 5 is tabulating the atomic feature involved in interactions while the graphical illustrations are given in the Fig 3.

**Table 5:** HPV E6 peptides–HLA-B*1501 and HLA-B*5801 interactions. FireDock energy for the best ranked complex initial distance between the H-bond donor and the acceptor; measured with the Find H.Bond tool in Chimera (H-Bond constraints were relaxed by 1 Å and 20.0 degrees) distance between the H-bond donor and the acceptor after molecular dynamics simulation (MD); measured in PyMOL, nd = no detected H-bond.

Peptide	Global Energy (kcal/mol)	vdW energy (kcal/mol)	H-Bond energy (kcal/mol)	Atomic interaction	
				Peptide-MHC atom pair	d_init_ (Å)
AT**LERTEVY**	**-30.47**	**-20.29**	**-1.36**	Asp61 O-Ala1 N	5.99
**HPV45** HLA-B*1501				Asp61 OD1-Ala1 N	5.68
				Asp61 OD2-Ala1 N	5.42
				Asp61 OD2-Glu4 N	5.12
				Arg62 NH2-Arg5 O	4.66
	
K**TLQRSE**VY	**-24.89**	**-18.84**	**-1.36**	Glu58 O-Lys1 NZ	4.03
**HPV58** HLA-B*1501				Tyr59 O-Lys1 NZ	5.80
				Asp61 OD2-Lys1 NZ	5.15
				Asp61 OD1-Thr2 OG1	6.37
				Asp61 OD2-Thr2 OG1	5.15
				Asp61 OD1-Arg5 NH1	5.50
				Asp61 O-Arg5 NH2	6.82
				Asp61 OD1-Arg5 NH2	5.13
	
K**TLQRSE**VY	**-21.19**	**-20.46**	**-2.67**	Asn66 O-Ser6 OG	4.74
**HPV58** HLA-B*5801				Asn66 OD1-Ser6 OG	4.10
				Ser70 OG -Ser6 OG	2.50
				Thr73 OG1-Ser6 OG	4.61
				Tyr99 OH -Ser6 O	3.19
				Ser70 OG -Ser6 OG	2.50
				Ser70 OG -Tyr9 O1	4.75
				Thr73 OG1-Ser6 OG	4.61
				Arg97 NH1-Leu3 O	5.85
				Arg97 NH1-Ser6 O	2.62
				TRP167 NE1-Tyr9 OH	5.88

### Molecular docking of E6 proteins with HLA-C*0801

**Fig 4:**
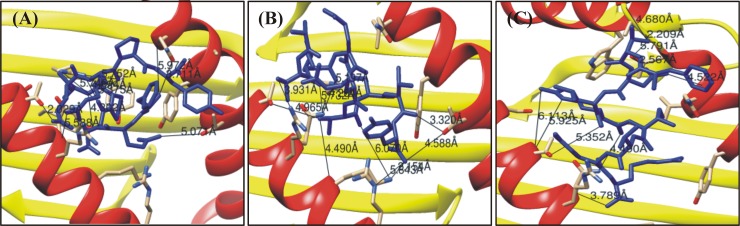
Molecular interaction analysis of predicted HPVs E6 peptides docked to MHC-I HLA-C*0801. Epitopes (A) FQDPAERPY, (B) ATLERTEVY and (C) FTDLRIVYR shown in the figure above was docked against HLA-C*0801.

Among the total epitopes, only three epitopes restricted to HLA-C*0801 were found to have the percentile rank below 2%. Docking of these peptides against MHC class I HLA-C*0801 revealed good atomic interactions. The interacting global energies and attractive van der Waals energy (vdW) were measured ranging from -15.97 to -30.36 kcal/mol and -22.99 to -24.88 respectively. These peptides were selected as specie specific. Arg97, Ser77, Asn114, Gln155 from the MHC-I groove were most abundantly involved in bonding with different predicted peptides. Post-docking analysis confirmed the stability of the complexes by observing the hydrogen bonding within 3Å. Variable number of hydrogen bonds from 2–4 with 3Å were found. This analysis established a good interaction of the modeled antigenic peptides with the MHC-I molecules. Molecular interactions of the specific peptides and their respective docked poses are represented in Fig 4. Molecular interactions of the specific peptides and their respective docked poses are tabulated in Table 6, while the structural complexes before and after simulation are given in the S5 Fig.

**Table 6:** HPV E6 peptides–HLA-C*0801 interaction. FireDock energy for the best ranked complex initial distance between the H-bond donor and the acceptor; measured with the Find H.Bond tool in Chimera (H-Bond constraints were relaxed by 1 Å and 20.0 degrees) distance between the H-bond donor and the acceptor after molecular dynamics simulation (MD); measured in PyMOL, nd = no detected H-bond.

Peptide	Global Energy (kcal/mol)	vdW energy (kcal/mol)	H-Bond energy (kcal/mol)	Atomic interaction	
				Peptide-MHC atom pair	d_init_ (Å)
**FQDPAERPY**	**-15.97**	**-22.99**	**-0.69**	Gln155 O-Phe1 N	5.97
**HPV35**				Gln155 OE1-Phe1 N	3.71
				Thr163 OG1-Gln2 NE2	5.07
				Arg69 O-Arg7 NH2	5.58
				Gln70 NE2-Arg7 O	5.37
				Thr73 OG1-Glu6 OE2	2.02
				Ser77 OG -Ala5 O	5.08
				Arg97 NH1-Asp3 O	4.89
				Arg97 NH1-Arg7 O	5.75
				Arg97 NH2-Asp3 O	5.74
				Asn114 ND2-Pro4 O	4.87
	
**ATLERTEVY**	**-35.17**	**-24.88**	**-1.74**	Thr73 OG1-Thr 6 OG1	4.96
**HPV45**				Tyr9 OH -Thr 6 O	4.41
				Arg62 NE -Tyr 9 OXT	5.84
				Lys66 NZ -Thr 6 O	6.07
				Arg69 NH2-Thr 2 OG1	3.93
				GLN70 NE2-Thr 2 O	5.73
				GLN70 NE2-Leu 3 O	4.26
				Thr73 OG1-Thr 6 OG1	4.96
				Arg97 NH1-Leu 3 O	5.19
				Tyr99 OH -Tyr 9 O	5.01
				Asn114 ND2-Glu 7 OE2	4.18
				Arg97 NH1-Glu7 OE2	2.35
				Lys66 NZ -Tyr9 OXT	2.15
				Arg97 NH1-Glu7 OE2	2.35
				Arg97 NH2-Glu7 OE2	3.57
				Lys66 NZ -Tyr9 O	3.07
				Lys66 NZ -Tyr9 OXT	2.15
	
FTDLRIVYR	**-30.36**	**-23.21**	**-0.92**	Thr152 OG1-Thr 2 OG1	5.79
**HPV52**				Thr73 O -Arg 5 NE	5.92
				Thr143 OG1-Arg 5 NH1	5.32
				Thr73 O -Arg 5 NH2	6.11
				Thr143 OG1-Arg 5 NH2	5.44
				Tyr99 OH -Arg 9 NH1	6.15
				Arg62 NH2-Val 7 O	6.01
				Gln70 N -Arg 9 O1	3.78
				Gln70 NE2-Arg 5 O	4.49
				Thr73 OG1-Leu 4 O	5.35
				Arg97 NH1-Arg 5 O	4.18
				Arg97 NH1-Tyr 8 O	5.19
				Arg97 NH2-Arg 5 O	4.98
				Arg97 NH2-Tyr 8 O	5.29
				Gln155 N -Asp 3 OD2	4.52

### Pharmacokinetics simulation based validation

**Fig 5:**
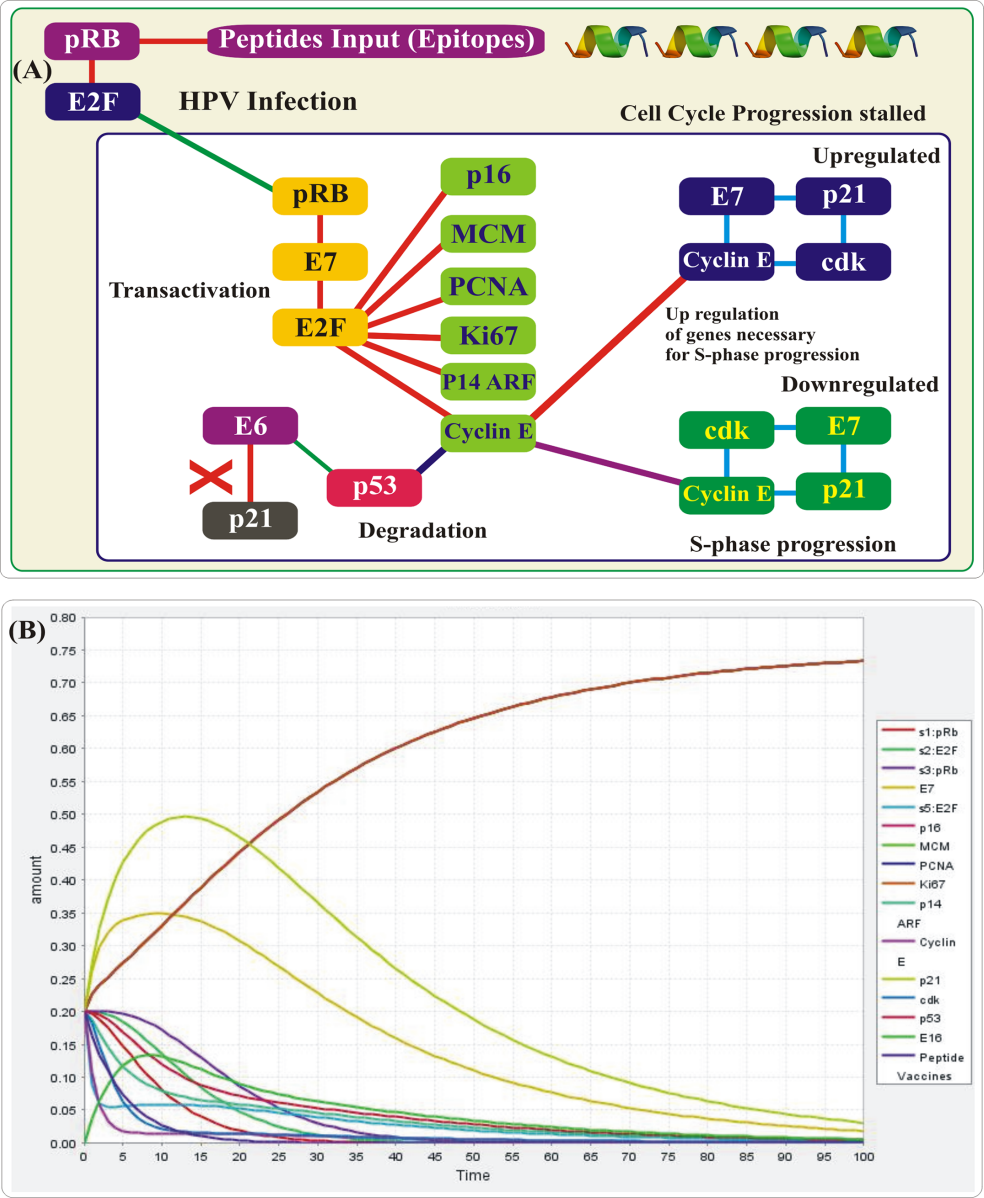
**(A)** Pharmacokinetics simulation showing the pathway of degrading the p53 genes in HPV infections, while in **(B)** X-axis represents the transition time of entities and Y-axis represent the concentration of the peptides.

**Fig 6:**
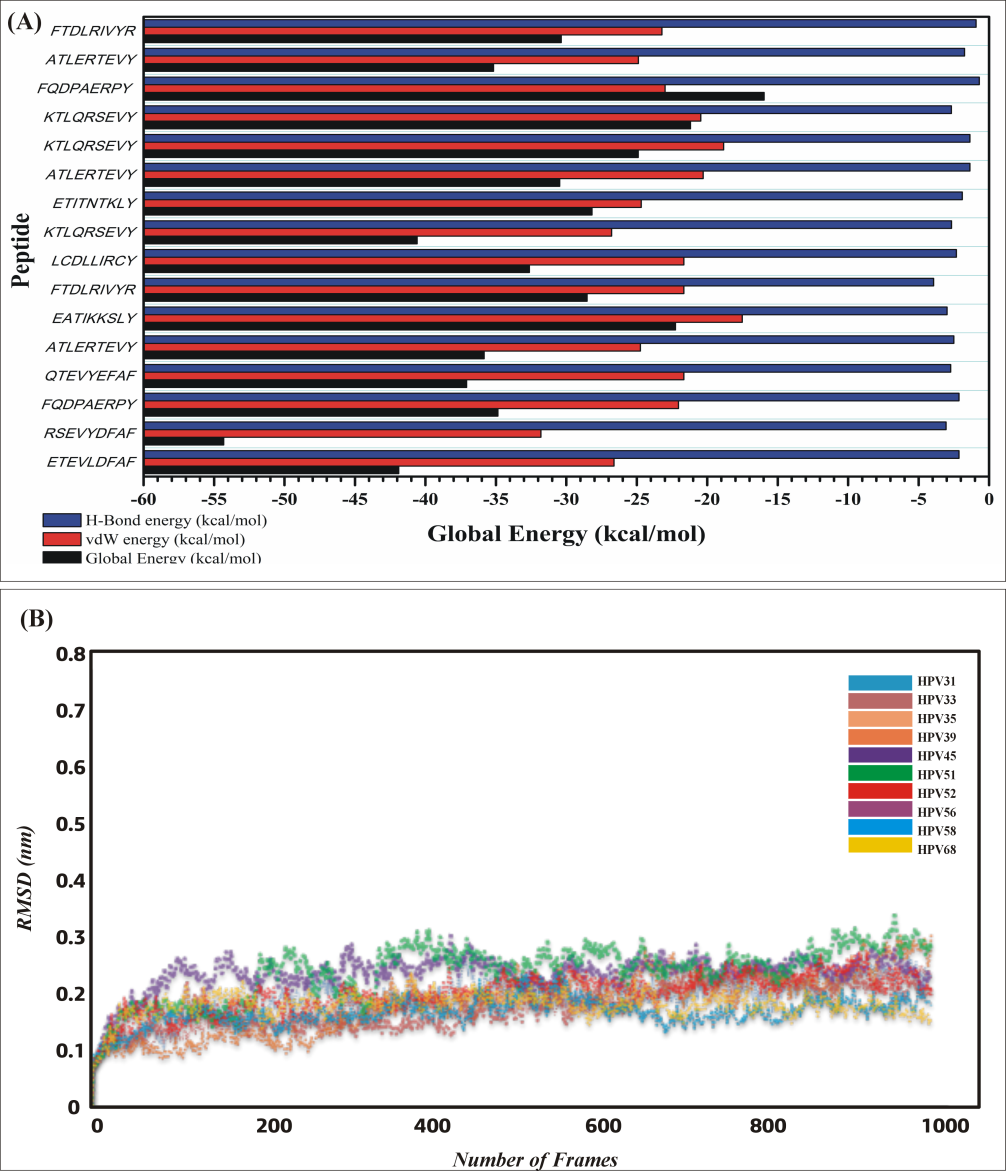
**(A)** The binding affinities (Global energies, vdW, electrostatic interactions) of all the docked epitopes are given in the graph. **(B)** Plot of RMSD of the backbone structures of MHCI-peptides complexes after 20ns simulation. The RMSD graph of each complex is shown in different colors. The graph (along x-axis is number of residues, y-axis RMSD in nm) is showing the stable movement of all the complexes.

Biological network representations have been used to explain interaction as well as mechanisms between entities, and the biochemical mathematical models of the network have been used to study the pharmacokinetic mechanism in the specific systems. Pharmacokinetics simulation, as shown in the Fig 5, reported that during High Risk HVPs Infection, pRb activates the E2F which infect E7, and combined E2F and E7 activates the E2F, which make basal, and parabasal layer and convert into transactivation which up regulate the genes necessary for S-phase progression. Up regulation of E7 and down regulation of p21 form E7+p21 complex, where p21 inactivated and Cyclin E/cdk present in activated form at low levels. While down regulation of E7 and up regulation of p21 form p21+E7+Cyclin E complex, where Cyclin E/cdk inactivated and present in activated form at high levels. Peptide inactivates the MDM through p14 ARF, which degrade p53. This mechanism of degrading the tumor suppressor genes MDM and p53 is already reported by the experimental studies and thus increasing the potential of our study [21,22]. Furthermore, we have used an experimentally reported concentration to obtain the similar results. The binding affinities of all the docked epitopes are given in the graph Fig 6(A).

### Structural stability of complexes

Stability of the complexes was examined by analyzing the trajectories from simulation. Root mean square deviation (RMSD) of the Cα atoms was calculated to analyze the stability using CPPTRAJ. The RMSD as shown in the Fig 6(B) of all the simulated systems are reaching the equilibrium is shorter and the averaged RMSD values of all the bound complexes range from 0.1 to 0.25 (nm). These results imply that the bound peptides restrain the motions of MHC-I and favor the stability of the complex structure.

### Epitopes cluster analysis

All of the predicted epitopes for hrHPVs E6 were grouped into 16 clusters as shown in the (S4 Table). Among the 16 hrHPVs’ E6 clusters, cluster 3 and 5 contain the most 7 epitopes out of the total selected 10. On the other, hrHPVs E6 clusters, cluster 4 contained the most epitopes (4 epitopes), while the rest of the clusters possess two and one epitopes respectively.

## Discussion

Vaccination is one of the significant approaches to improve the standard of community health, provide the best and safe way to control the increasing infections leading to complex diseases. Modern therapeutics developments greatly rely on immunoinformatics resources for the synthesis of antigen-specific epitopic therapeutic vaccines against the wide diversity of pathogenic infections [34,59]. Using these *in silico* methods, highest accuracy is reported by different applied groups. Epitopic vaccine against HIV, malaria and tuberculosis provided promising results and supported the defensive and therapeutic uses of these vaccines [60].

Genomic and proteomic information of Human Papillomaviruses delineate that E6 and E7 proteins has significant therapeutic value that could be targeted to prevent the progression of HPVs persistent infection. Targeting E6 protein is important for the immunotherapy of cancer as these are reported to be important in the survival and maintenance of oncoproteins in the malignant cell [61–63]. The present study predicted epitopes for twelve different MHC class I HLAs to provide wide spectrum of peptides from HPVs E6 proteins that could strongly provoke the immune response. Detail analysis of the predicted epitopes such as percentile rank, MHC binding affinity, TAP, C-terminal cleavage activity, antigenicity and allergenic profiling was carried out to select the most promising epitopes as this criteria is experimentally validated for immune response potential [64–66]. The epitopes predicted in this study could become a clinical candidate sooner or later for the treatment of HPVs infection and cervical cancer, as epitopes such as KLPQLCTEL_18-26_ and FAFRDLCIV_52-60_ of E6 proteins, have been tested in a transgenic mice for IC_50_ value which resulted in immune response in experimental conditions [67]. Previous studies already verified that peptide FAFRDLCIVYR_52-62_ possess antitumor effect and is reported to be processed by T-cell endogenously [68,69]. The current study predicted epitopes for the E6 proteins, which also contain regions from epitope, such as FAFRDLCIVYR_52-62_, thus increasing the accuracy and validating our study to be taken to the experimental room. Furthermore, we tested our predicted epitopes RREVYDFAF_46-54_ (Cluster 3) for the optimal peptide properties, using PepCalc, which resulted in promising water solubility and other good experimental properties. Our results showed that combining cluster 3 and cluster 4 will form a continues epitope that will elicit robust immune response against multiple species of HPVs thus acting as more active prophylactic vaccine candidate, as present in seven species, for the clinical exploration. Previous study also reported that the combined epitopes PYAVCDKCLKF_66-76_ of the E6 protein presented by HLA-A*1101 and HLA-A*2402 with a worldwide population frequency ranging 29.5–30.5% possess positive behavior. As a result, the epitope combinations we predicted (Cluster 5 _65–75_) might be feasible in therapeutic vaccines of hrHPVs [70]. The purpose of our study was to predict and select T-cell epitopes because they are more promising and evoke a long-lasting immune response, and because with antigenic drift, an antigen can easily escape the memory response of antibody. However, in vaccine development, allergenicity is a prominent hurdle [71]. Today, most vaccines stimulate the immune system into an ‘allergic’ reaction, through induction of type 2 T helper cells and immunoglobulin E. Therefore, we also confirmed the non-allergenic potential of our validated epitopes. The allergenicity testing results of these epitopes showed that our epitopes are safe and will not show any allergenic reaction possibly. Furthermore, Docking, MD simulations and PKPD modeling approach also verified that our peptides has experimental potential.

The current state of the study strongly support the development of peptide based vaccines against the HPVs species that are active and has largest number of infections but neglected. To date only one or two species of HPVs are studied but no such large meta-analysis integrated with dynamics is reported. This meta-analysis of multiple therapeutically important species has increased the scope and accuracy of this study. Our results specified the regions from 46 to 54, 65 to 76 and a combined epitope from 46 to 62 could be used for development of candidate CTL epitopes. To overcome the worldwide distribution of the major epidemic of hrHPVs, the predicted epitopes restricted to different HLAs could cover most of the vaccination and desire outcome. This study aided the development of prophylactic vaccines that will provide cross immunity against multiple cervical causing species.

## Conclusion

In the current study, we utilized advanced immunoinformatics and molecular dynamics simulations approaches to predict and verify potential T-cell epitopes to act as vaccine candidates against the hrHPVs. Many previous studies conveyed different information only about the major HPV16, 18 and HPV45 but none of the study focused the other important performers of cervical cancer. We have disclosed a wide range of information and predicted potential vaccine agents that will act against multiple species of cervical cancer causing agents. Prediction, docking, post docking and simulation analysis will aid the development of prophylactic vaccines against hrHPVs. The advantage of this study is that it covers many aspects along with multiple risk species of cervical cancer. However, further experimental insight will be fruitful in taking the predicted potential peptides into the clinical room and market.

## Supporting information

S1 Table The detail information accession number, individual protein sequence length and region etc. are shown in the table below. (DOCX)

S2 Table The table is showing the predicted minimum and maximum surface hydrophilicity score of E6 proteins of hrHPVs. (DOCX)

S3 Table The table is showing the predicted minimum and maximum surface hydrophilicity score of E6 proteins of hrHPVs. (DOCX)

S4 Table Cluster analysis of all epitopes of hrHPVs E6 predicted. (DOCX)

S1 Fig The figure is showing the graphical representation of predicted antigenic propensity of E6 proteins of hrHPVs. Predicted epitope residue positions are colored in yellow.
(PDF)

S2 Fig The figure is showing the graphical representation of predicted surface accessibility of E6 proteins of hrHPVs. The red line is showing the default threshold. Yellow color residue is showing residues above the threshold.
(PDF)

S3 Fig The figure is showing the graphical representation of predicted surface flexibility of E6 proteins of hrHPVs. The red line is showing the default threshold. Yellow color residue is showing residues above the threshold.
(PDF)

S4 Fig The figure is showing the graphical representation of predicted surface hydrophilicity of E6 proteins of hrHPV. The red line is showing the default threshold for Surface Hydrophilicity prediction. Yellow color residue is showing residues with Surface Hydrophilicity above the threshold.
(PDF)

S5 Fig hrHPVs E6 peptide-MHC-I protein complexes (cartoon representation); the complex after MD Simulation 20-ns (in cyan) is superimposed with the complex before the MD simulation (in green): (A) ETEVLDFAF (HPV31), (B) RSEVYDFAF (HPV33), (C) FQDPAERPY (HPV35), (D) QTEVYEFAF (HPV39), (E) ATLERTEVY (HPV45), (F) FTDLRIVYR (HPV52), (G) LCDLLIRCY (HPV56), (H) KTLQRSEVY (HPV58).
(PDF)
