# Supplementary material for: Computational identification, characterization and validation of potential antigenic peptide vaccines from hrHPVs E6 proteins using immunoinformatics and computational systems biology approaches
Source: PLoS One. 2018 May 1;13(5):e0196484. doi: 10.1371/journal.pone.0196484 (PMC5929558; doi:10.1371/journal.pone.0196484)
Supplement: S1 Table — (DOCX) [file pone.0196484.s001.docx]

**Table S1.** The detail information accession number, individual protein sequence length and region etc. are shown in the table below.

| **S. No** | **Species** | **Uniprot Accession Number** | **Protein Name** | **No of Amino Acids** |
| --- | --- | --- | --- | --- |
| 1. | HPV31 | P17386 | E6 | 149AA |
| 2. | HPV33 | P06427 | E6 | 149AA |
| 3. | HPV35 | P24835 | E6 | 149AA |
| 4. | HPV39 | P27228 | E6 | 158AA |
| 5. | HPV45 | P21735 | E6 | 158AA |
| 6. | HPV51 | P26554 | E6 | 151AA |
| 7. | HPV52 | P36814 | E6 | 149AA |
| 8. | HPV56 | P24836 | E6 | 155AA |
| 9. | HPV58 | Q547M1 | E6 | 149AA |
| 10. | HPV68 | P54667 | E6 | 158AA |
