# Supplementary material for: Computational identification, characterization and validation of potential antigenic peptide vaccines from hrHPVs E6 proteins using immunoinformatics and computational systems biology approaches
Source: PLoS One. 2018 May 1;13(5):e0196484. doi: 10.1371/journal.pone.0196484 (PMC5929558; doi:10.1371/journal.pone.0196484)
Supplement: S2 Table — (DOCX) [file pone.0196484.s002.docx]

**Table S2.** The table is showing the predicted minimum and maximum Surface Hydrophilicity score of E6 Proteins of hrHPVs.

| **Species** | **Position** | **Residue** | **Score** | **Residues Score >1** |
| --- | --- | --- | --- | --- |
| **HPV31** | 7 | **E** | 1.598 | 12 |
|  | 100 | **L** | -2.26 |  |
| **HPV33** | 7 | **E** | 1.737 | 64 |
|  | 66 | **C** | -2.554 |  |
| **HPV35** | 7 | **E** | 1.615 | 63 |
|  | 100 | **L** | -2.786 |  |
| **HPV39** | 9 | **E** | 1.461 | 65 |
|  | 105 | **C** | -2.871 |  |
| **HPV45** | 9 | **Q** | 1.893 | 75 |
|  | 102 | **L** | -2.473 |  |
| **HPV51** | 111 | **G** | 1.586 | 67 |
|  | 66 | **C** | -2.002 |  |
| **HPV52** | 7 | **T** | 1.756 | 62 |
|  | 66 | **C** | -2.813 |  |
| **HPV56** | 10 | **E** | 1.907 | 50 |
|  | 69 | **C** | -2.584 |  |
| **HPV58** | 7 | **E** | 1.625 | 49 |
|  | 102 | **R** | -2.5 |  |
| **HPV68** | 9 | **E** | 1.617 | 48 |
|  | 102 | **L** | -2.313 |  |
