# Supplementary material for: Computational identification, characterization and validation of potential antigenic peptide vaccines from hrHPVs E6 proteins using immunoinformatics and computational systems biology approaches
Source: PLoS One. 2018 May 1;13(5):e0196484. doi: 10.1371/journal.pone.0196484 (PMC5929558; doi:10.1371/journal.pone.0196484)
Supplement: S3 Table — (DOCX) [file pone.0196484.s003.docx]

**Table S3.** The table is showing the predicted minimum and maximum Surface Hydrophilicity antigenic propensity score of E6 Proteins of hrHPVs.

| **Species** | **Position** | **Residue** | **Peptide** | **Score** | **Residues Score >1** |
| --- | --- | --- | --- | --- | --- |
| **HPV31** | 132 | **W** | GGR**W**TGR | 0.081 | 97 |
|  | 31 | **V** | LNC**V**YCK | 1.189 |  |
| **HPV33** | 143 | **R** | WRS**R**RRE | 0.893 | 106 |
|  | 103 | **C** | ILIR**C**II | 6.371 |  |
| **HPV35** | 132 | **W** | GGR**W**TGR | 0.081 | 103 |
|  | 30 | **C** | CLN**C**VYC | 1.258 |  |
| **HPV39** | 147 | **E** | TKR**E**DRR | 0.882 | 113 |
|  | 110 | **K** | CCL**K**PLC | 1.247 |  |
| **HPV45** | 154 | **R** | RRR**R**ETQ | 0.895 | 109 |
|  | 32 | **C** | SIA**C**VYC | 1.228 |  |
| **HPV51** | 145 | **Q** | RTR**Q**RNE | 0.881 | 101 |
|  | 30 | **C** | QVV**C**VYC | 1.307 |  |
| **HPV52** | 132 | **W** | MGR**W**TGR | 0.875 | 100 |
|  | 31 | **V** | LQC**V**QCK | 1.202 |  |
| **HPV56** | 4 | **Q** | MEP**Q**FNN | 0.914 | 118 |
|  | 68 | **V** | VCR**V**CLL | 1.28 |  |
| **HPV58** | 132 | **W** | SGR**W**TGR | 0.901 | 109 |
|  | 64 | **K** | AVC**K**VCL | 1.262 |  |
| **HPV68** | 148 | **D** | KRE**D**RRR | 0.877 | 114 |
|  | 53 | **C** | SDL**C**VVY | 1.21 |  |
