# Supplementary material for: Computational identification, characterization and validation of potential antigenic peptide vaccines from hrHPVs E6 proteins using immunoinformatics and computational systems biology approaches
Source: PLoS One. 2018 May 1;13(5):e0196484. doi: 10.1371/journal.pone.0196484 (PMC5929558; doi:10.1371/journal.pone.0196484)
Supplement: S4 Table — (DOCX) [file pone.0196484.s004.docx]

**Table S4.** Cluster analysis of all epitopes of hrHPVs E6 predicted.

| **Cluster Number** | **Number of Epitopes in the Cluster** | **Epitope Number** | **Epitope Name** | **Epitope Sequence** |
| --- | --- | --- | --- | --- |
| 1 | 1 | 1 | 1 | LSSALEIPY |
| 2 | 1 | 1 | 2 | LTETEVLDF |
| 3 | 7 | 1 | 3 | ETEVLDFAF |
|  |  | 2 | 6 | RSEVYDFAF |
|  |  | 3 | 10 | QTEVYEFAF |
|  |  | 4 | 18 | RTEVYQFAF |
|  |  | 5 | 21 | RADVYNVAF |
|  |  | 6 | 29 | RSEVYDFVF |
|  |  | 7 | 34 | RTEVYEFAF |
| 4 | 4 | 1 | 4 | FTDLTIVYR |
|  |  | 2 | 12 | FSDLYVVYR |
|  |  | 3 | 23 | FTDLRIVYR |
|  |  | 4 | 35 | FSDLCVVYR |
| 5 | 7 | 1 | 5 | VSEFRWYRY |
|  |  | 2 | 7 | ISEYRHYNY |
|  |  | 3 | 9 | ISEYRWYRY |
|  |  | 4 | 13 | YAKIRELRY |
|  |  | 5 | 19 | YSRIRELRY |
|  |  | 6 | 24 | ISEYRHYQY |
|  |  | 7 | 26 | YSKVRKYRY |
| 6 | 1 | 1 | 8 | FQDPAERPY |
| 7 | 1 | 1 | 11 | YEFAFSDLY |
| 8 | 1 | 1 | 14 | YSDSVYATT |
| 9 | 2 | 1 | 15 | NTKLYNLLI |
|  |  | 2 | 20 | NTELYNLLI |
| 10 | 1 | 1 | 16 | CTELNTSLQ |
| 11 | 2 | 1 | 17 | ATLERTEVY |
|  |  | 2 | 28 | KTLQRSEVY |
| 12 | 2 | 1 | 22 | EAITKKSLY |
|  |  | 2 | 37 | ETITNTKLY |
| 13 | 2 | 1 | 25 | YRHYQYSLY |
|  |  | 2 | 32 | YRHYNYSLY |
| 14 | 1 | 1 | 27 | LCDLLIRCY |
| 15 | 1 | 1 | 30 | RLLSKISEY |
| 16 | 1 | 1 | 33 | TLDTTLHDV |
