# Supplementary figures and images for: Computational identification, characterization and validation of potential antigenic peptide vaccines from hrHPVs E6 proteins using immunoinformatics and computational systems biology approaches
Source: PLoS One. 2018 May 1;13(5):e0196484. doi: 10.1371/journal.pone.0196484 (PMC5929558; doi:10.1371/journal.pone.0196484)

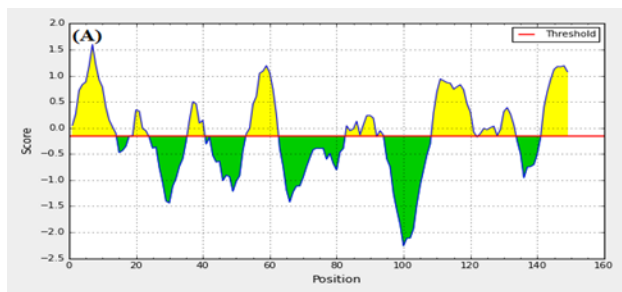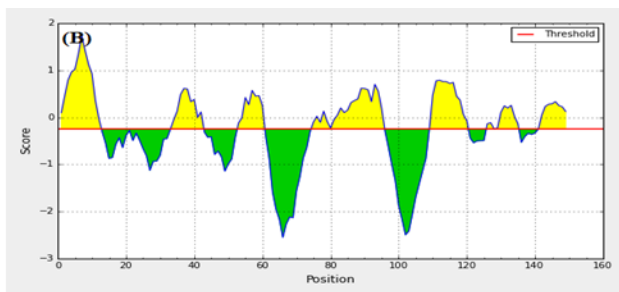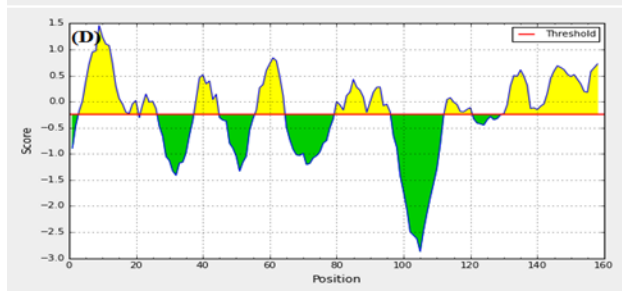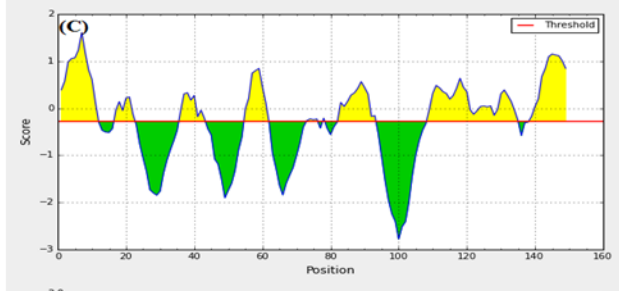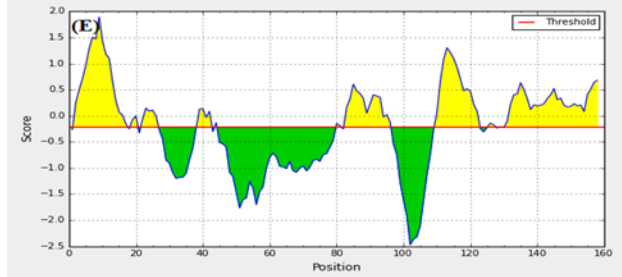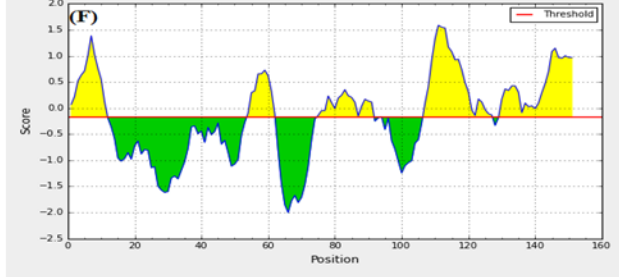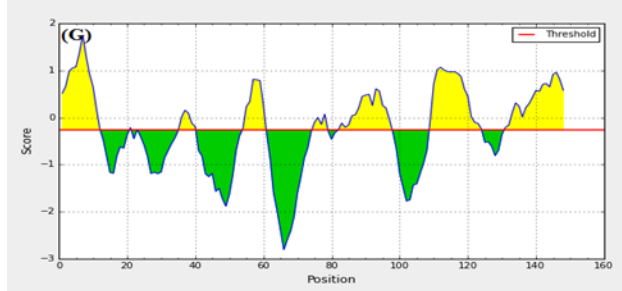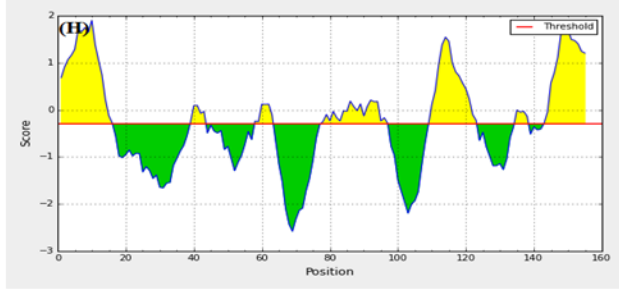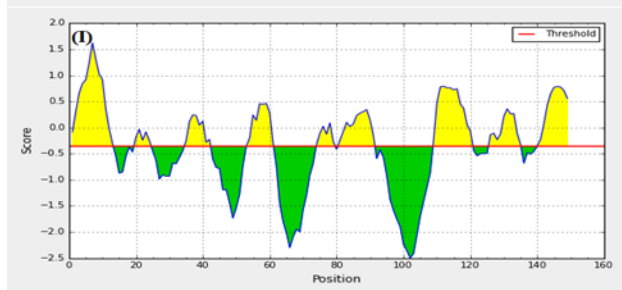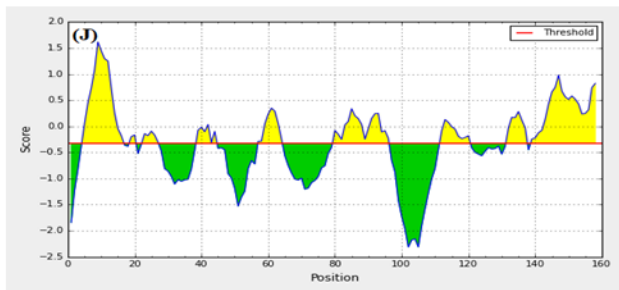

Supplement: S1 Fig — Predicted epitope residue positions are colored in yellow. (PDF) [file pone.0196484.s005.pdf]

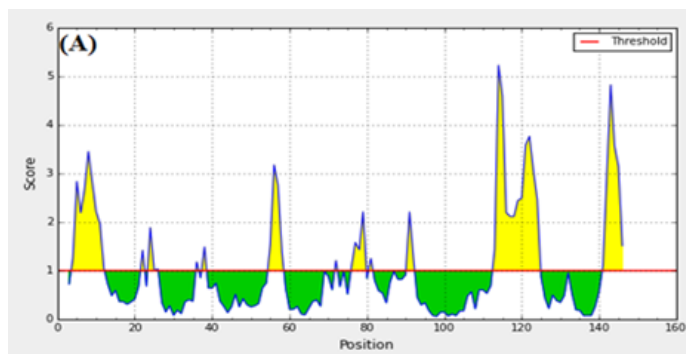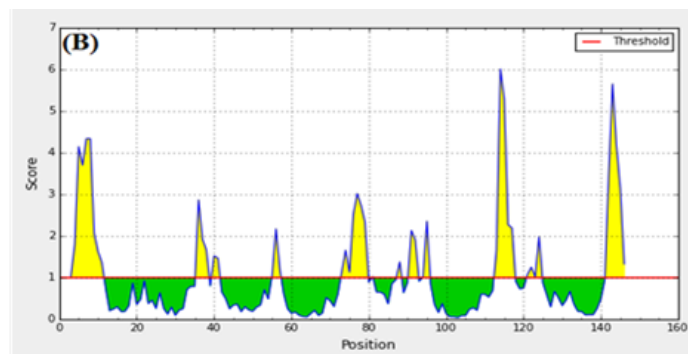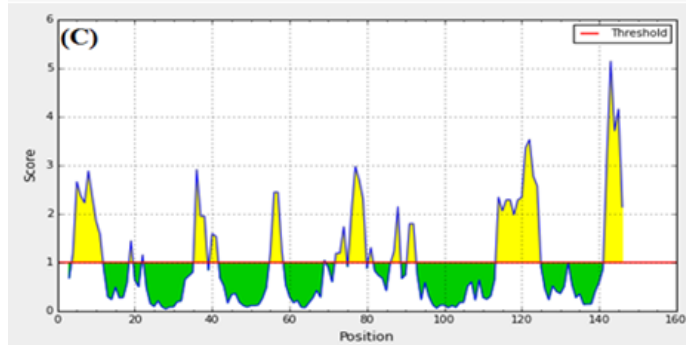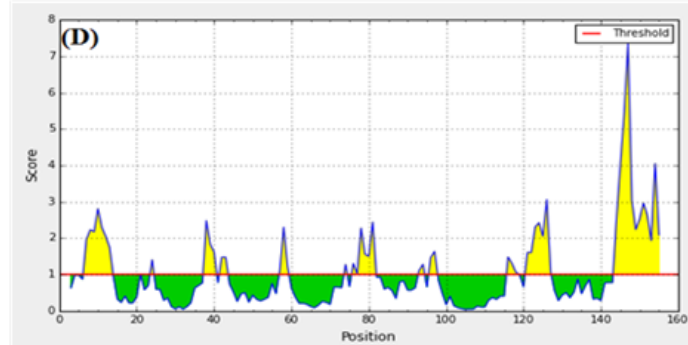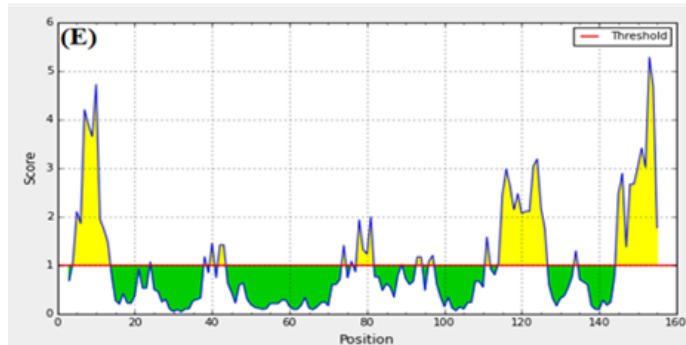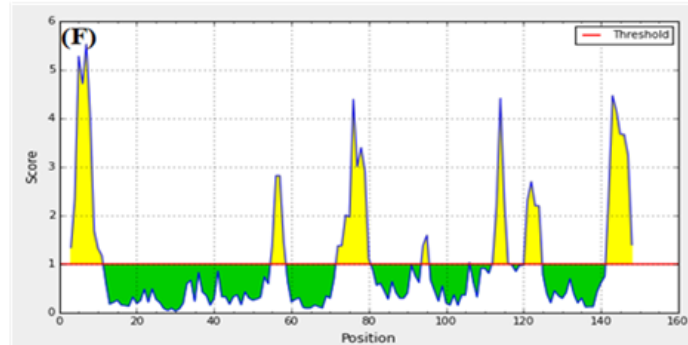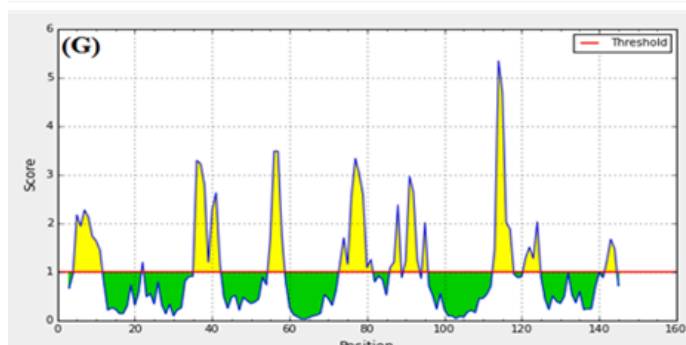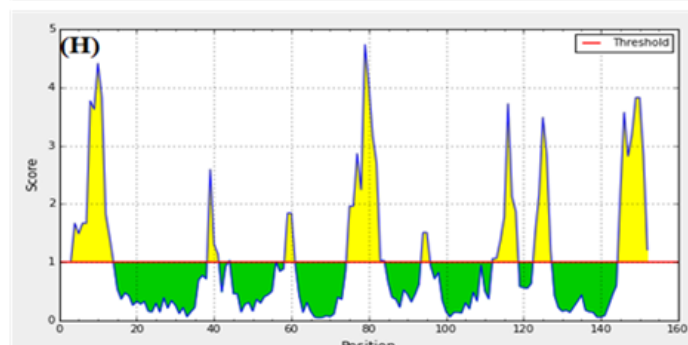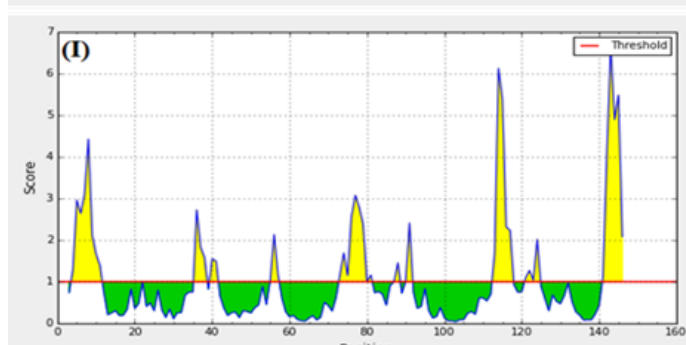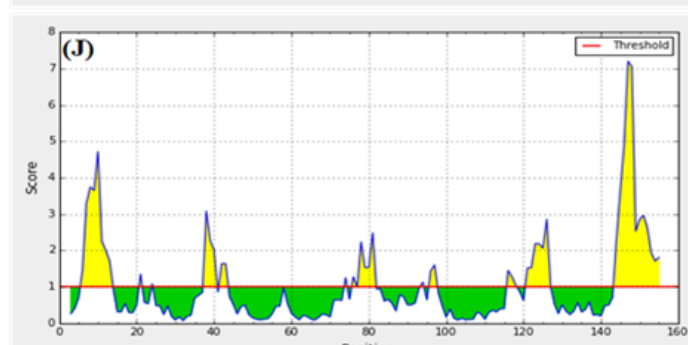

Supplement: S2 Fig — The red line is showing the default threshold. Yellow color residue is showing residues above the threshold. (PDF) [file pone.0196484.s006.pdf]

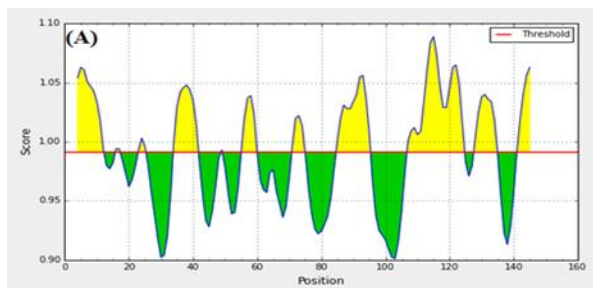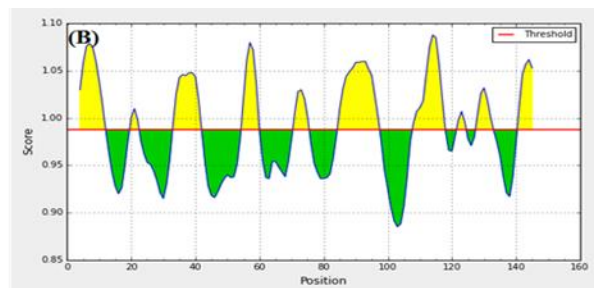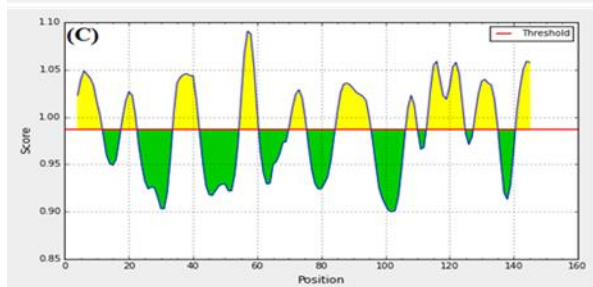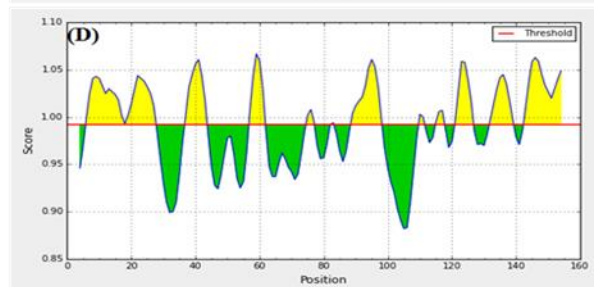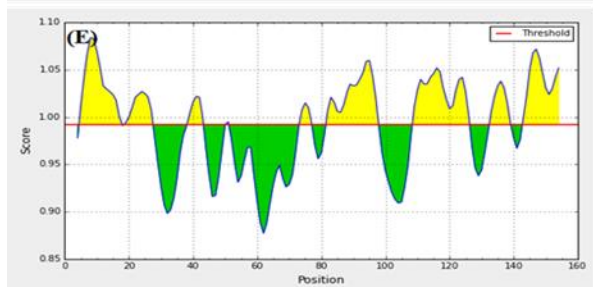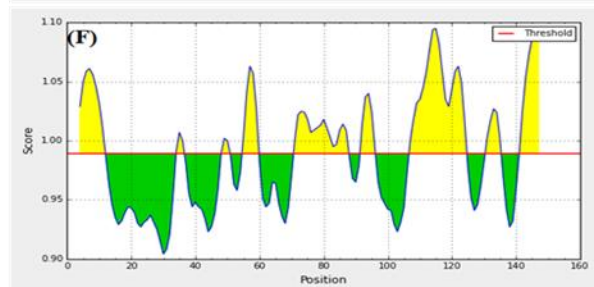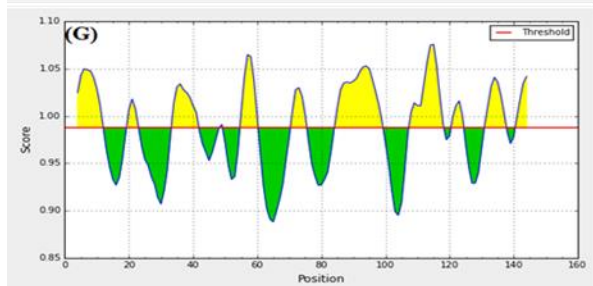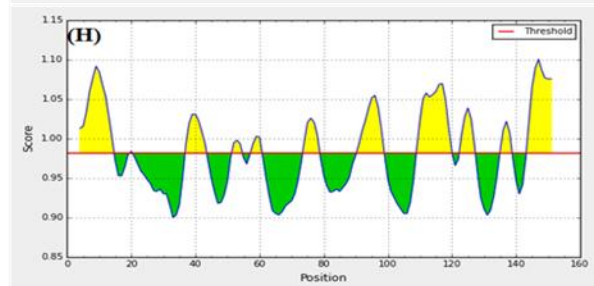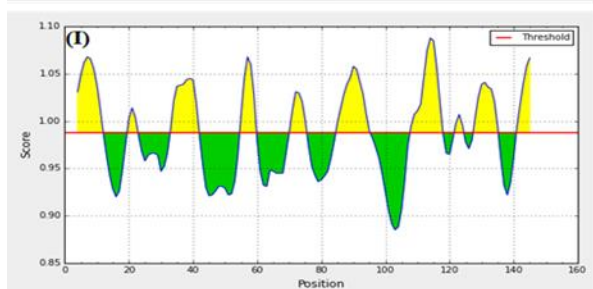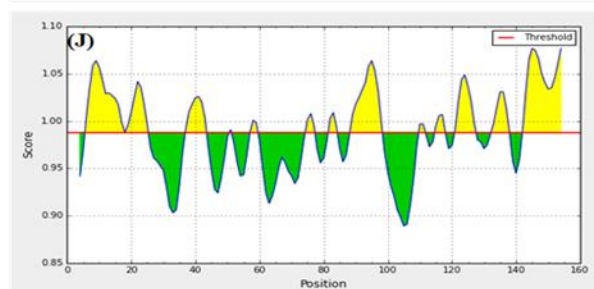

Supplement: S3 Fig — The red line is showing the default threshold. Yellow color residue is showing residues above the threshold. (PDF) [file pone.0196484.s007.pdf]

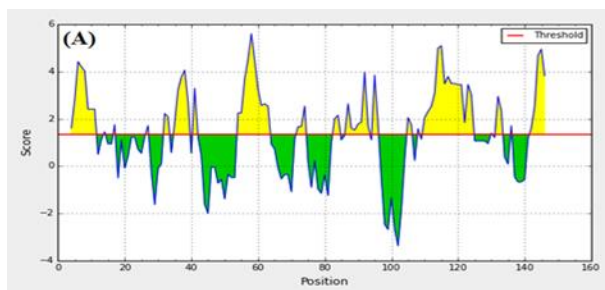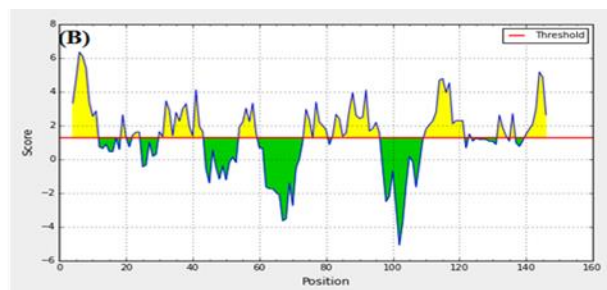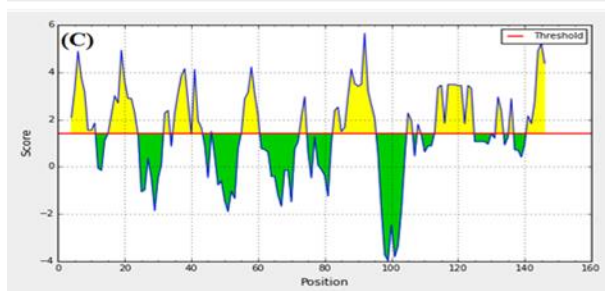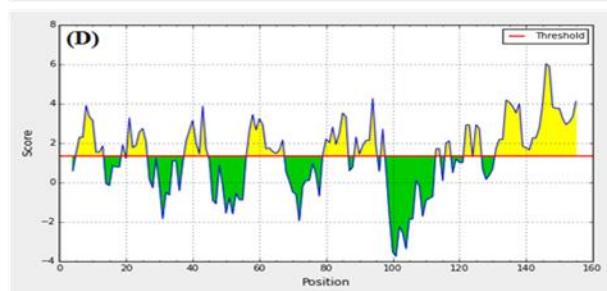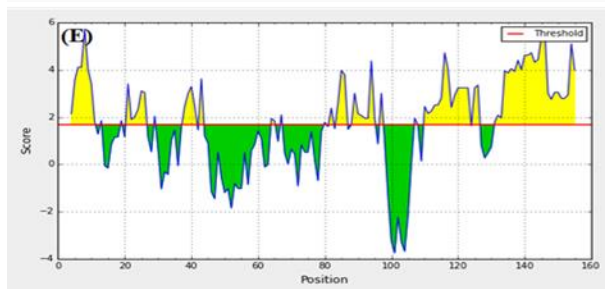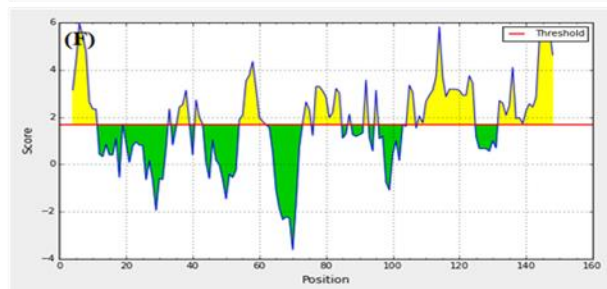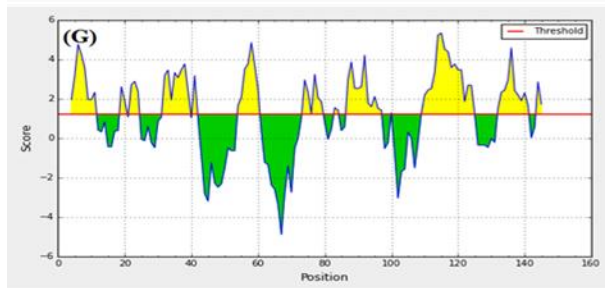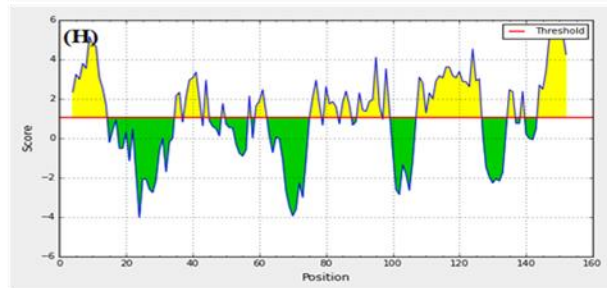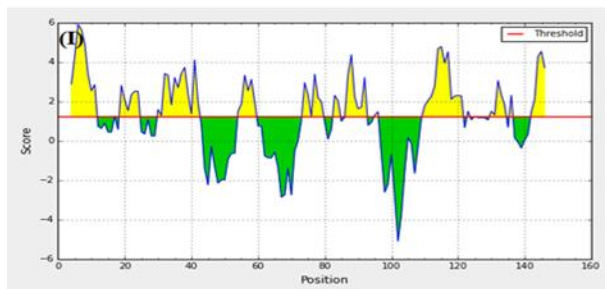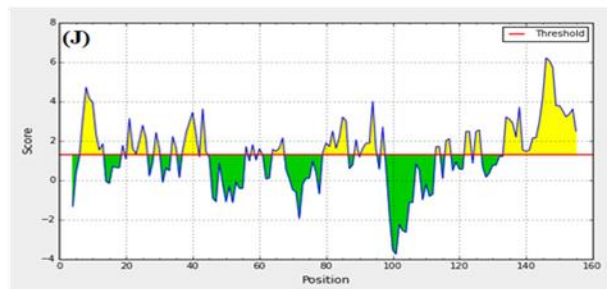

Supplement: S4 Fig — The red line is showing the default threshold for Surface Hydrophilicity prediction. Yellow color residue is showing residues with Surface Hydrophilicity above the threshold. (PDF) [file pone.0196484.s008.pdf]

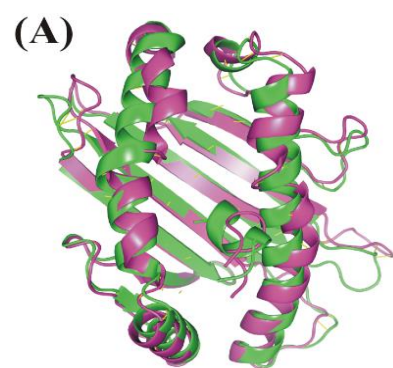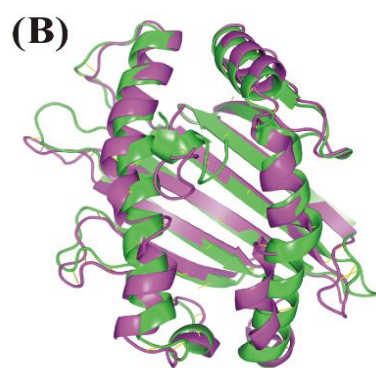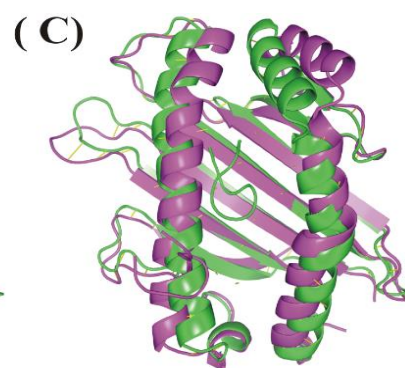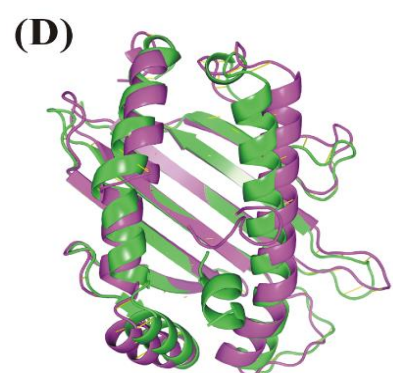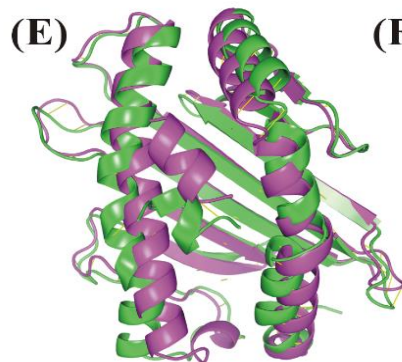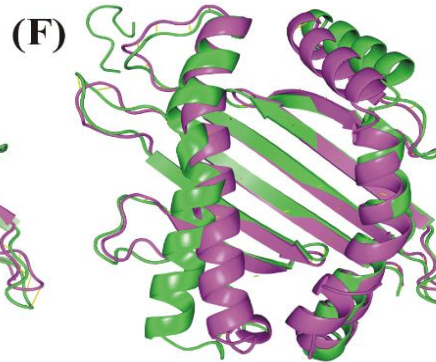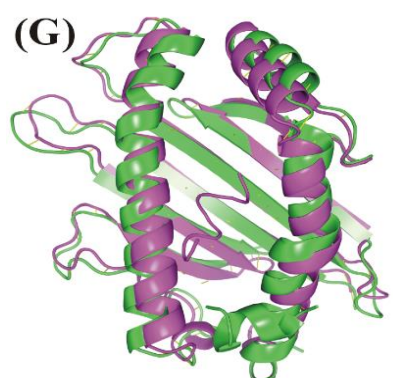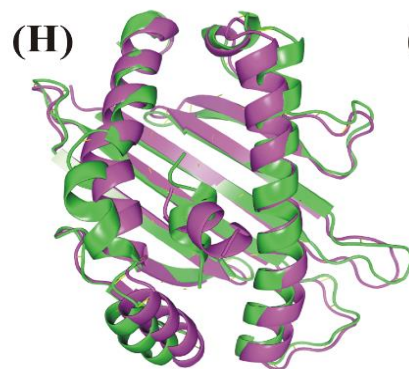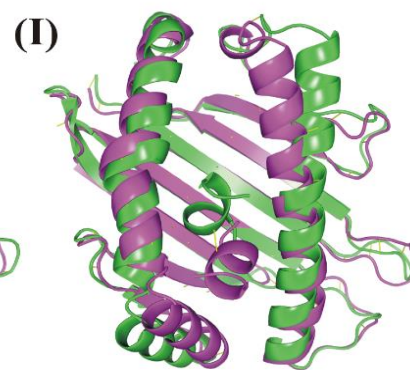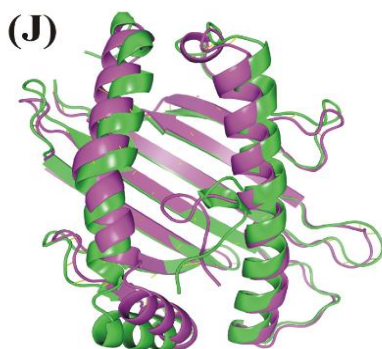

Supplement: S5 Fig — hrHPVs E6 peptide-MHC-I protein complexes (cartoon representation); the complex after MD Simulation 20-ns (in cyan) is superimposed with the complex before the MD simulation (in green): (A) ETEVLDFAF (HPV31), (B) RSEVYDFAF (HPV33), (C) FQDPAERPY (HPV35), (D) QTEVYEFAF (HPV39), (E) ATLERTEVY (HPV45), (F) FTDLRIVYR (HPV52), (G) LCDLLIRCY (HPV56), (H) KTLQRSEVY (HPV58). (PDF) [file pone.0196484.s009.pdf]
